# Comparative DNA methylomic analyses reveal potential origins of novel epigenetic biomarkers of insulin resistance in monocytes from virally suppressed HIV-infected adults

**DOI:** 10.1186/s13148-019-0694-1

**Published:** 2019-06-28

**Authors:** Christian K. Dye, Michael J. Corley, Dongmei Li, Vedbar S. Khadka, Brooks I. Mitchell, Razvan Sultana, Annette Lum-Jones, Cecilia M. Shikuma, Lishomwa C. Ndhlovu, Alika K. Maunakea

**Affiliations:** 1 Department of Native Hawaiian Health, University of Hawaii John A. Burns School of Medicine, 677 Ala Moana Blvd, Suite 1016B, University of Hawaii, Honolulu, HI 96813 USA; 2 Department of Tropical Medicine, University of Hawaii, 651 Illalo Street, BSB 325C, Honolulu, HI 96815 USA; 3 Department of Molecular Bioscience and Bioengineering, University of Hawaii, 1955 East-West Road, Ag. Science 208, Honolulu, HI 96822 USA; 4 Hawaii Center for AIDS, University of Hawaii, 651 Ilalo St. BSB 231, Honolulu, HI 96813 USA; 5 Office of Biostatistics & Quantitative Health Sciences, University of Hawaii John A. Burns School of Medicine, 651 Ilalo Street, Medical Education Building, Suite 411, Honolulu, HI 96813 USA; 6 School of Medicine and Dentistry, University of Rochester Medical Center, 601 Elmwood Ave., CU430708, Rochester, NY 14642 USA

**Keywords:** Monocyte, Insulin resistance, Diabetes, DNA methylation, Epigenetics, Inflammation, Immune response, Cardiometabolic disease, HIV

## Abstract

**Background:**

Compared to healthy individuals, those with stably repressed HIV experience a higher risk of developing insulin resistance, a hallmark of pre-diabetes and a major determinant for cardiometabolic diseases. Although epigenetic processes, including in particular DNA methylation, appear to be dysregulated in individuals with insulin resistance, little is known about where these occur in the genomes of immune cells and the origins of these alterations in HIV-infected individuals. Here, we examined the genome-wide DNA methylation states of monocytes in HIV-infected individuals (*n* = 37) with varying levels of insulin sensitivity measured by the homeostatic model assessment of insulin resistance (HOMA-IR).

**Results:**

By profiling DNA methylation at single-nucleotide resolution using the Illumina Infinium HumanMethylation450 BeadChip in monocytes from insulin-resistant (IR; HOMA-IR ≥ 2.0; *n* = 14) and insulin-sensitive (IS; HOMA-IR < 2.0; *n* = 23) individuals, we identified 123 CpGs with significantly different DNA methylation levels. These CpGs were enriched at genes involved in pathways relating to glucose metabolism, immune activation, and insulin-relevant signaling, with the majority (86.2%) being hypomethylated in IR relative to IS individuals. Using a stepwise multiple logistic regression analysis, we observed 4 CpGs (*cg27655935*, *cg02000426*, *cg10184328*, and *cg23085143*) whose methylation levels independently predicted the insulin-resistant state at a higher confidence than that of clinical risk factors typically associated with insulin resistance (i.e., fasting glucose, 120-min oral glucose tolerance test, Framingham Risk Score, and Total to HDL cholesterol ratio). Interestingly, 79 of the 123 CpGs (64%) exhibited remarkably similar levels of methylation as that of hematopoietic stem cells (HSC) in monocytes from IR individuals, implicating epigenetic defects in myeloid differentiation as a possible origin for the methylation landscape underlying the insulin resistance phenotype. In support of this, gene ontology analysis of these 79 CpGs revealed overrepresentation of these CpGs at genes relevant to HSC function, including involvement in stem cell pluripotency, differentiation, and Wnt signaling pathways.

**Conclusion:**

Altogether, our data suggests a possible role for DNA methylation in regulating monocyte activity that may associate with the insulin-resistant phenotype. The methylomic landscape of insulin resistance in monocytes could originate from epigenetic dysregulation during HSC differentiation through the myeloid lineage. Understanding the factors involved with changes in the myeloid trajectory may provide further insight into the development of insulin resistance. Furthermore, regulation of specific genes that were implicated in our analysis reveal possible targets for modulating immune activity to ameliorate insulin resistance.

**Electronic supplementary material:**

The online version of this article (10.1186/s13148-019-0694-1) contains supplementary material, which is available to authorized users.

## Introduction

Cardiometabolic disorders, including type 2 diabetes mellitus (T2D) and cardiovascular disease, are among the most prevalent and debilitating diseases in the US and worldwide. Notably, despite stable antiretroviral therapy (ART), individuals infected with HIV are at a higher risk than non-infected individuals for developing chronic non-infectious illness traditionally associated with older age including cardiometabolic diseases [1] and cognitive dysfunction [2], reminiscent of aging-associated inflammation, or “inflamm-aging.” Prior studies have suggested that inflammation may cause this increase in non-AIDS mortality and morbidity [3, 4]. Higher levels of interleukin-6 (IL-6), C-reactive protein (CRP), D-dimer, solubleCD163 (a monocyte activation marker), and type 1 interferon (IFN)-α responses were observed in HIV patients on ART compared to non-infected individuals from the general population [5–7]. In addition, HIV-infected individuals are at a significantly higher risk of developing insulin resistance [8, 9], a hallmark of pre-diabetes and a major determinant for the onset of T2D [10, 11]. Evidence is emerging for the role of inflammation in the pathogenesis of insulin resistance [12]. TNF-α was observed to be increased in adipose tissue and correlated with insulin resistance; ablating TNF-α was shown to decrease insulin resistance in animal models [13]. Although inflammation and insulin resistance are key risk factors in cardiometabolic diseases [14], irrespective of HIV infection status, the regulation of immune cell activity underlying cardiometabolic disease risk remains incompletely understood.

Evidence is emerging for the role of epigenetic processes, particularly DNA methylation, in inflammation and insulin resistance [15]. Inflammation has been shown to be associated with global DNA hypermethylation in peripheral blood mononuclear cells (PBMCs) [16] and with insulin resistance, independent of other risk factors for T2D [17]. Interestingly, differential DNA methylation at specific genomic loci in PBMCs exhibited changes at genes related to immune function and inflammatory pathways, which are associated with biomarkers of inflammation [18]. A prospective study examining PBMCs from T2D patients and matched non-diabetics observed DNA methylation differences at a few loci that were associated with T2D risk [19]. Another study found that DNA methylation variable positions preceded T2D diagnosis [20]. Some of these local changes in DNA methylation that are associated with inflammation appeared to be reversible by surgical treatments of obesity, which restored insulin sensitivity [21]. Thus far, most studies have examined DNA methylation differences in T2D almost exclusively in insulin-sensitive organs, including pancreatic islets, skeletal muscle, and hepatic tissue [22]. Elucidating DNA methylation differences in peripheral inflammatory cells whose states may be altered in individuals at risk for developing cardiometabolic diseases, i.e., insulin resistant, was therefore of interest. To our knowledge, only one study has sought to determine the epigenetic signature of insulin resistance; however, this was observed in adipose tissue and bulk populations of PBMCs [23]. The heterogeneity in cell type composition of PBMCs makes it difficult to attribute the methylation changes to a particular cell type that may underlie inflammation and insulin resistance phenotypes.

Monocytes play a central role in both acute and chronic inflammation, forming one of the first lines of defense against pathogens through phagocytosis, antigen presentation, and cytokine production. In one study, nutritionally induced reversal in the inflammatory states of monocytes from T2D patients was observed [24], suggesting monocyte function and related inflammation may be under epigenetic regulation. Monocytes develop initially in the bone marrow, emigrate into peripheral blood where they provide routine immunosurveillance, responding to infection/inflammation, and migrate into tissues where they differentiate into macrophages [25]. Monocytes display proinflammatory features, secreting various inflammatory cytokines (i.e., tumor necrosis factor alpha [TNF-α], IL-6, interleukin 1-beta [IL-1β] and interleukin-8 [IL-8]) after stimulation with toll-like receptor (TLR) ligands [26]. TNF-α is an inflammatory cytokine involved in systemic inflammation that is induced by lipopolysaccharide (LPS), other bacterial products, and IL-1 s. CD16-expressing monocytes are a major source of TNF-α [27]. *TNF-α* gene expression is induced by high glucose treatment of monocytes [28] and neutralization of TNF-α improves insulin sensitivity in an animal model of T2D [13]. Obesity, another risk factor for insulin resistance, induces alterations in monocytes and macrophages, stimulating the production of chemokines and cytokines (i.e., monocyte chemoattractant protein-1 [MCP-1/CCL2], IL-6, IL8, IL-1β, TNF-α), and leads to insulin resistance in target cells by activating c-jun N-terminal kinase (JNK) and I-Kappa-B-Kinase-Beta (IKKβ/NF-κB) pathways [12, 29]. Monocytes from HIV-infected individuals exhibited higher variability in immune responses as measured by the production of proinflammatory cytokines compared to non-infected individuals [30], prompting us to examine whether this variability may underlie their heightened risk for insulin resistance. The transcriptional levels of proinflammatory cytokines are regulated by DNA methylation [31]. Together, these studies suggest that the DNA methylation landscape of monocytes may be altered in monocytes of insulin-resistant individuals, possibly exhibiting a higher degree of variability than that of insulin-sensitive individuals.

Additionally, we recently reported an immunoepigenetic signature of cognitive impairment in monocytes from HIV-infected individuals with dementia [32], prompting us to examine whether altered DNA methylation states in these cells may also relate to insulin resistance and cardiometabolic disease risk. Thus, we evaluated genome-wide DNA methylation states in HIV-infected individuals (*n* = 37) with varying levels of insulin sensitivity, measured by the homeostatic model assessment of IR (HOMA-IR) using fasting insulin and glucose levels. As expected, individuals diagnosed as insulin resistant (IR; HOMA-IR≥2.0) had a higher risk for cardiovascular disease as measured by the Framingham Risk Score (FRS) than their insulin-sensitive (IS; HOMA-IR < 2.0) counterparts [33]. IR individuals also exhibited unique DNA methylation differences enriched at genes involved in signaling pathways, including inflammatory and glucocorticoid signaling. These associations are likely independent of HIV, as we observed a 3% overlap of differentially methylated CpGs in monocytes of HIV-infected and non-infected in our comparative analyses of insulin resistance. These results support a recent study describing an association between DNA methylation states at specific loci in T cells with IR in non-infected individuals [34]. Furthermore, our data implicates defects in myeloid differentiation as a potential origin of the monocyte methylation states associated with the IR phenotype.

## Materials and methods

### Sample cohorts and clinical data

This prospective study was conducted utilizing data and specimens selected from the first two years of the Hawaii Aging with HIV Cohort-Cardiovascular Disease (HAHC-CVD) study, a 5-year natural history study investigating the role of chronic HIV infection in the development of cardiovascular disease (CVD) among HIV-infected participants on suppressive ART. Inclusion criteria from the cohort of 160 patients required documented HIV-positive status, use of combination ART ≥ 6 months, male, and non-diabetic at time of blood draw. Extensive HIV immunologic and cardiometabolic assessments were available from this cohort, and additional characteristics have been previously described [35]. Viably cryopreserved PBMCs were obtained from the Hawaii Center for AIDS (HICFA). Clinical and immunological parameters for each individual available, included age, gender, ethnicity, total cholesterol, high-density lipoprotein- cholesterol (HDL-c), low-density lipoprotein cholesterol (LDL-c) smoking status, systolic blood pressure, use of anti-hypertensive medication, viral load (HIV RNA), CD4+ cell counts, diabetic status, HOMA-IR, body mass index (BMI), insulin sensitivity, fasting glucose, oral glucose tolerance test (OGTT), and FRS (Table 1). Of the 160 patients in the cohort, we excluded diabetic subjects and females due to sex differences affecting genome-wide DNA methylation and gene expression in pancreatic β-islets and association with insulin secretion [36], and limited sample size. Finally, due to exhaustion of available PBMCs from the cohort, 37 individuals were selected for inclusion in the study. These 37 individuals were stratified into one of two groups on the basis of their HOMA-IR scores, calculated using fasting blood glucose and insulin levels as a surrogate measure of β cell function and insulin sensitivity: HOMA-IR < 2.0 was considered insulin sensitive (IS; *n* = 23), whereas a HOMA-IR ≥ 2.0 was considered insulin resistant (IR; *n* = 14) based on comparisons to previous studies [37–39]. Informed consent was obtained from participants following procedures approved by the University of Hawaii Human Studies Program (CHS #16476). All experiments were performed in accordance with relevant guidelines and regulations.

**Table 1:**
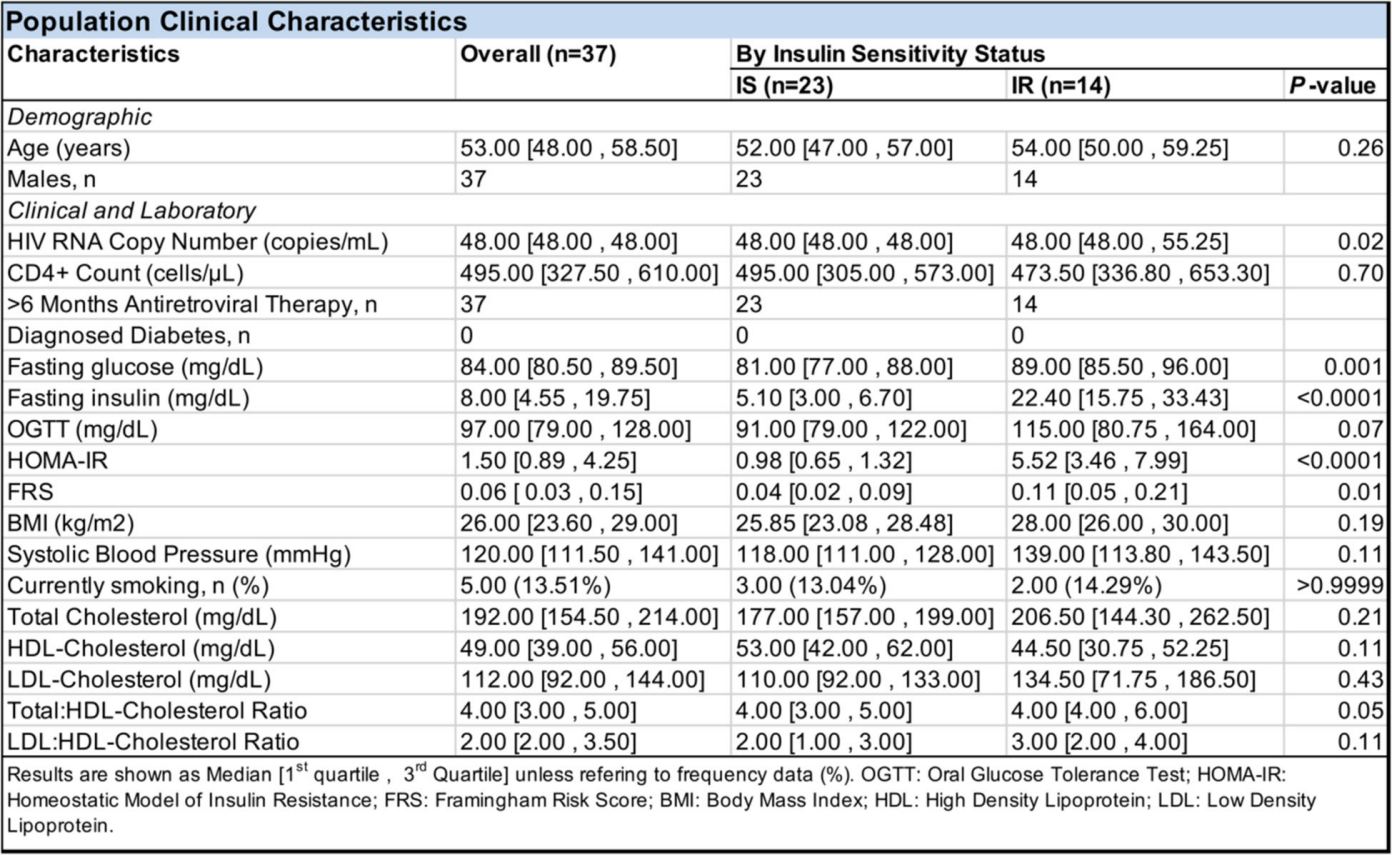
Baseline characteristics of insulin-sensitive (IS) and insulin-resistant (IR) individuals

Clinical data comparing the IS and IR group. Data shown are median values [first quartile , third quartile], except for frequency counts (%). *P* value was determined between IS and IR groups using Mann-Whitney *U* test; significance at *P* < 0.05

### Measuring systemic levels of inflammation in plasma

To evaluate systemic cytokine/chemokine protein levels, we performed a custom 8-plex bead-based multiplexing assay using the Milliplex Human Cytokine/Chemokine Magnetic Bead Panel following the manufacturer’s recommendations (Millipore, Darmstadt, Germany) on plasma biospecimens from IR and IS individuals acquired during the same blood draws for PBMC. Antibodies for interferon gamma (IFN-γ), interleukin-10 (IL-10), IL-1β, IL-6, IL-8, TNF-α, MCP-1, and vascular endothelial growth factor (VEGF) were utilized in this custom 8-plex panel. Standards, controls 1 and 2, and samples were measured in duplicates. The human cytokine standards were customized to include a lower than recommended concentration (0.64 pg/mL) to increase the range of the standard curve for lower limits of detection. To further improve sensitivity of the assay for analytes, the plates were incubated overnight at 4 °C for 17 hours with constant agitation. Fluorescent signals were analyzed using the Luminex 200™ instrument (R&D Systems, Inc., Minneapolis, MN, USA). Bio-Plex Manager™ software (Bio-Rad Laboratories, Inc., Hercules, CA, USA) was used for data processing.

### PBMC specimens, monocyte enrichment, and nucleic acid isolation

Viably cryopreserved PBMCs of approximately 2.5 × 10^6^ to 1.7 × 10^7^ from HIV-infected individuals were first thawed in AIM-V Serum Free Media (Thermo Fisher Scientific, Inc., Waltham, MA, USA) supplemented with 1:50 DNase (Sigma-Aldrich, St. Louis, MO, USA), washed, and resuspended in wash buffer (PBS, 3% BSA, and 1 mM EDTA). Aliquots of 1.25 × 10^5^ cells (PBMCs) were taken from each sample prior to enrichment for flow cytometry-based cellular phenotyping assays to determine cell type composition. Monocytes were enriched from PBMCs using the Negative-Selection, Human Monocyte Enrichment Kit without CD16 Depletion (StemCell Technologies, Inc, Vancouver, BC, Canada) following the manufacturer’s guidelines utilizing the purple EasySep™ magnet (StemCell Technologies). Cells were counted prior to cell separation using the Countess® Automated Cell Counter (Life Technologies™, Carlsbad, CA, USA) to determine the minimal cell concentration required for cell:antibody/magnetic bead binding necessary for monocyte enrichment. Negatively selected cells were counted again after monocyte enrichment to partition the appropriate number of cells required for flow cytometry (1.0 × 10^5^–1.25 × 10^5^ cells) to confirm efficiency of monocyte enrichment, while the remainder of enriched cells were pelleted and resuspended in lysis buffer for subsequent purification of nucleic acids. DNA and RNA were isolated from enriched monocytes using the AllPrep® DNA/RNA Mini Kit (Qiagen, Hilden, Germany) according to the manufacturer’s recommendations for purification of DNA and RNA from animal cells. Nucleic acid concentrations were quantified using the Qubit® 2.0 Fluorometer (Thermo Fisher Scientific) following the manufacturer’s protocol. Qubit® dsDNA HS Assay Kit and Qubit® RNA BR Assay Kit (Thermo Fisher Scientific) were used for DNA and RNA, respectively. DNA and RNA were stored in − 20 °C and − 80 °C, respectively.

### Validation of monocyte enrichment by flow cytometry

To confirm the enrichment of monocytes by negative selection, an aliquot of PBMCs (1.25 × 10^5^ cells) for all subjects pre- and post-enrichment was analyzed by a flow cytometer for monocytes, T cells, NK cells, and B cells (Table 2). Aliquots were stained with yellow amine fluorescent reactive dye (YARD; Thermo Fisher Scientific) then with anti-CD16 Brilliant Violet 421 (Clone 3G8), anti-CD3 V500 (Clone UCHT1), anti-CD14 Qdot®605 (Clone TüK4), anti-CD56 Pe-Cy7 (Clone B159), anti-CD19 PE-Cy7 (Clone SJ25C1), anti-CD20 Pe-Cy7 (Clone 2H7), and anti-HLA-DR APC-H7 (Clone G46-6) for identification of leukocyte subpopulation frequencies. Anti-CD16 was purchased from BioLegend, Inc., San Diego, CA, USA. Anti-CD3, anti-CD56, anti-CD20, anti-CD19, and anti-HLA-DR were obtained from BD Bioscience, San Jose, CA, USA. Anti-mouse Ig/Negative Control (FBS) Compensation Particle Set (BD Bioscience) was used for compensation analysis of fluorescent signals emitted by each fluorochrome from the multi-colored cellular phenotyping panel employed. Anti-mouse Ig compensation beads were stained with each fluorochrome-conjugated antibody in separate wells. ArC™ Amine Reactive Compensation Bead Kit (ThermoFisher Scientific) reactive bead/negative beads were used for compensation of YARD (Live/Dead stain) fluorescent signals. Stained cells from PBMCs, enriched monocytes, and compensation particles were analyzed using a 4-laser BD LSRFortessa flow cytometer (BD Bioscience). The data was analyzed using the FlowJo software (Tree Star, Inc., Ashland, OR, USA). The frequency (%) of each cell type was determined by event count (specific event/total events) with debris exclusion. Successful enrichment was observed for all samples in each group with an average monocyte enrichment of 89.5% (87.5%, 92.8%) and 88.7% (85.2%, 95.9%) for the IS and IR groups, respectively (shown: median [first quartile, third quartile]; Table 2). High-quality enrichment was necessary to diminish background noise caused by mixed cell populations, as the heterogeneity of cell populations confounds downstream DNA methylation analyses [40].

**Table 2:**
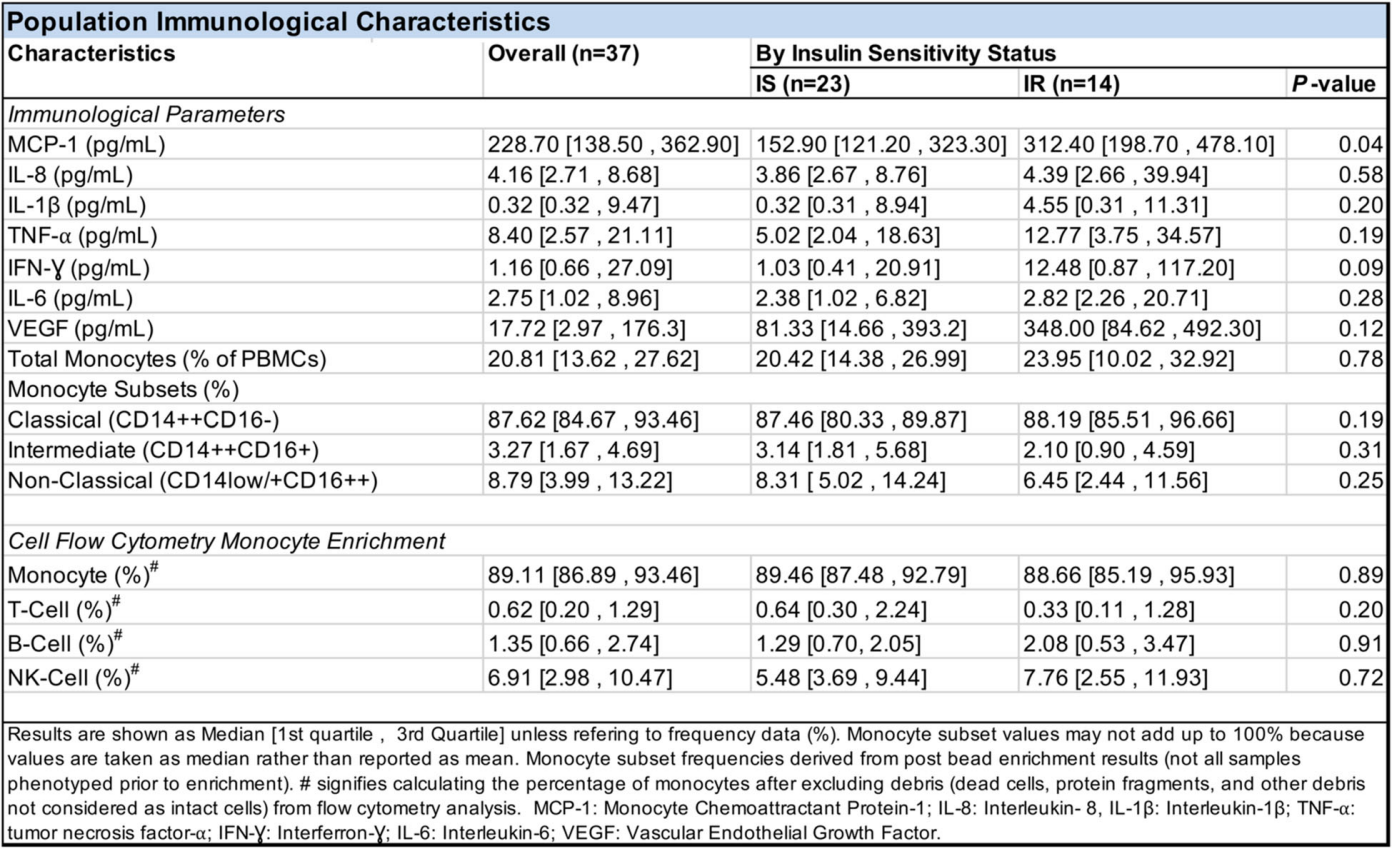
Overall and HOMA-IR stratified baseline immunological and cell flow cytometry characteristics of study cohort

Immunological data, including cytokine/chemokine data derived from Luminex assays and monocyte composition data derived from flow cell cytometry assays. Data shown are median values [first quartile, third quartile]. Differences between IS and IR groups for each parameter shown were determined by Mann-Whitney *U* test and resulting *P* values are shown with significance at *P* < 0.05

### Illumina 450 K array-based DNA methylation analysis

The Illumina Infinium HumanMethylation450 BeadChip (450 K; Illumina®, Inc., San Diego, CA, USA) is capable of quantifying DNA methylation at over 450,000 cytosine-guanine dinucleotides (CpG) distributed throughout the genome at single-nucleotide resolution based on the ratio of fluorescent intensities between the methylated and unmethylated alleles of each CpG locus. To perform the 450 K microarray using the Illumina® Infinium® HD Methylation assay (Illumina®, Inc.), a minimum of 500 ng of genomic DNA from isolated monocytes was first treated with sodium bisulfite using the EZ DNA Methylation™ Kit (Zymo Research, Irvine CA, USA) following Illumina’s suggested protocol. Illumina iScan SQ scanner was utilized for chip imaging to receive intensities of hybridized CpG probes. Preprocessing was performed on raw IDAT data files with RNBeads 0.99.10 R [41]. Methylation data (*β* values; 0.00–1.00, as a percent from unmethylated to methylated) was normalized using methylumIDAT and Subset-quantile within array normalization (SWAN) to reduce technical variations on the microarray. Of the ~ 450,000 CpG sites that were quantified for methylation, a total of ~ 15,000 probes were removed after filtering for missing probes, SNP-enriched probes, and probes with statistically insignificant low intensity detections (*P* > 0.05), leaving 435,267 CpGs. From those remaining, we sought to identify sites with significant DNA methylation differences (*δ* of the *β* value) between the IS and IR groups (*P* < 0.05) using the resampling-based empirical Bayes Methods permutation approach with *P* < 0.05 [42] and filtered for sites with absolute average methylation differences greater than 10% (*δ*) between the IR and IS groups. This approach reduces the false discovery rates for non-normally distributed array-based data and offers higher statistical power. Incorporating differences at a threshold of 10% absolute difference in DNA methylation also diminishes the likelihood of random technical errors from true biological differences, increasing the confidence in detecting differences in DNA methylation [43]. We consider these CpGs as differentially methylated loci (DMLs). Gplot Bioconductor package was used to generate heatmaps of DMLs. Unsupervised hierarchical clustering was performed using Manhattan distances. Region enrichment analysis of distribution of DMLs for gene location and CpG island region was performed using chi-square goodness of fit tests with each category vs. sum of all other categories with Bonferroni correction. Similar to the above approach, we performed an analysis comparing methylation states of the DML from the IR or IS groups to that of the methylation states within hematopoietic stem cells (HSCs) (*n* = 3; GEO Accession: GSE87197) [44] and stratified differences between the IR or IS group and that of HSCs as CpGs maintaining methylation between HSCs and IR or IS individuals (*δ <* 10%). Power analysis using estimated differences and standard deviations in DNA methylation changes between groups was conducted to examine the power of our study. The results of this power analysis showed with a total sample size of at least 23 individuals, we have 80% confidence (false discovery rate = 0.05) in detecting an effect. Thus, this study was sufficiently powered.

### Cell type-specific differential methylation validation of monocyte enrichment

Illumina Infinium HumanMethylation450 BeadChip data was downloaded from GEO Accession: GSE35069 [45]. Cell type-specific methylation data for fluorescence-activated cell sorted (FACS) monocytes (*n* = 6) and PBMCs (*n* = 6) were used to determine cell type-specific DNA methylation sites using the resampling-based empirical Bayes Methods permutation approach as performed above but with an average methylation *δ* > 30% between monocytes and PBMCs [42]. This produced 5,124 CpGs whose methylation states appeared robustly cell type-specific, allowing distinction between monocytes and PBMCs derived from DNA methylation. We then compared the methylation states at these sites distinguishing PBMCs from monocytes to that from the monocyte methylation data of each of our samples to determine the degree of difference or similarity using a correlation analysis. Similarly, to determine if there was an enrichment of specific monocyte subpopulations (i.e., classical, intermediate, or non-classical monocytes), we developed our own means of identifying subsets based on cell type-specific DNA methylation, as previously performed in the monocyte-specific DNA methylation profiling [45]. Leukocytes were obtained by means of leukapheresis from two non-infected, healthy volunteers, and monocyte subsets were collected using FACS performed by the Flow Cytometry Services of the University of Hawaii at Manoa, John A. Burns School of Medicine (University of Hawaii, Honolulu, HI, USA). Monocyte subsets were then subjected to the 450 K microarray for methylation profiling and differentially methylated sites between each subset was determined by the resampling-based empirical Bayes Methods permutation approach as performed above with an average *δ* > 10% [42]. This revealed 394 CpGs, 128 CpGs, and 496 CpGs whose methylation states robustly stratified classical, intermediate, and non-classical monocytes, respectively. This molecular approach for determining cell identity complemented flow cytometry-based phenotype data, thereby increasing confidence in the efficiency of bead separation technology for enrichment of monocytes and their purity. DNA methylation-based approaches further allows for determining cellular identity, which goes beyond the limits of proteins displayed on the surface of cells of interest. A list of DNA methylation datasets retrieved from GEO that were utilized in this study can be found in Additional file 7: Table S1.

### Gene Ontology analysis

Gene ontology (GO) analysis was performed using Ma’ayan Lab’s Enrichr publicly available analysis program (http://amp.pharm.mssm.edu/Enrichr/), which utilizes specific single nucleotide positions of each CpG of interest to determine the nearest gene(s) each CpG is located at and the functional relevance these regions/genes may have to specific molecular, cellular, or biological processes and various pathways [46, 47]. GO analysis included annotation of differentially methylated loci (DML) between IR and IS, and DML that maintained DNA methylation levels between IS/IR and HSCs. Statistical significance of GO results were determined using Fisher’s exact test; significance at *P* < 0.05.

### Statistical analysis

Comparative analyses of clinical, immunological, cell flow cytometry, and DNA methylation data were performed using Mann-Whitney *U* test. Non-parametric tests for clinical, immunological, and cell flow cytometry data were used primarily due to the majority of data being non-normally distributed, as measured using the D’Agostino and Pearson test for normality. DNA methylation data was statistically analyzed using non-parametric tests due to the heteroscedastic nature of *β* values [43]. Overall, utilizing non-parametric statistical analyses reduces bias that parametric tests would confer and decreases the false discovery rate. Regional enrichment analysis of CpGs at specific gene locations and CpG island regional distribution was performed using chi-square goodness of fit. Spearman’s rho (*r* correlation coefficient) was used in statistical analysis for all associations tested. To predict associative outcomes, several stepwise multiple logistic regression models were tested on clinical features, immunological features, and differentially methylated sites that were identified by Wilcoxon Rank-Sum tests followed by multiple testing corrections (FDR-adjusted *P* < 0.01). Graphing and statistical analysis were performed using Prism 7, Version 7.0c (GraphPad, La Jolla, CA, USA). All statistical significance was determined at *P* < 0.05.

## Results

### Higher variability of clinical features associated with cardiometabolic disease is characteristic of insulin resistance

Clinical characteristics from individuals stratified into the IS or IR groups are shown in Table 1. As expected, the clinical factors defining insulin resistance by HOMA-IR (i.e., fasting glucose and insulin) were significantly higher among IR individuals compared to IS individuals (*P* < 0.05). In addition, we observed higher variability in clinical parameters (i.e., fasting insulin, HOMA-IR, total cholesterol, LDL-cholesterol, total to HDL cholesterol ration, and LDL to HDL-choleseterol ratio) among the IR group. The higher degree of intra-individual clinical variability in the IR group could be attributed to the increased risk of individuals in this group for developing cardiometabolic disease [48]. Indeed, Framingham Risk Scores (FRS), a measure of risk for developing cardiovascular disease, was significantly higher among the IR group compared to the IS group (*P* = 0.01; Table 1) consistent with prior observations [49–51]. Although HIV RNA copy number was significantly increased in the IR group (*P* = 0.02; Table 1), due to two outlier individuals having HIV RNA copy number above the threshold for undetectability (48 copies/mL), it was still within the threshold to be considered successfully suppressed (< 200 copies/mL). We next measured the association between HOMA-IR and clinical features typically associated with cardiometabolic disease risk. HDL cholesterol was inversely correlated to HOMA-IR; lower HDL cholesterol levels correlated to higher HOMA-IR (*r* = − 0.41; *P* = 0.01; Additional file 1: Figure S1A). Furthermore, the ratio of total cholesterol to HDL cholesterol concentration, a measurement used to assess risk of developing heart disease, was significantly correlated to HOMA-IR (*r* = 0.34; *P* = 0.04; Additional file 1: Figure S1B). Obesity, as measured by body mass index (BMI), is another independent risk factor for developing insulin resistance, as an increase in adiposity underlies mechanisms contributing to IR pathogenesis [52]. Consistent with this notion, we observed that individuals with higher BMIs also had greater HOMA-IR scores (*r =* 0.41; *P* = 0.02; Additional file 1: Figure S1C). We also observed a significant positive correlation between between FRS and HOMA-IR (*r* = 0.48; *P* = 0.003; Additional file 1: Figure S1D). Taken together, these results are consistent with prior observations that FRS, the ratio of total cholesterol to HDL cholesterol, and BMI are subclinical indicators of cardiometabolic disease risk [53–55]. We note that these data are representative of a larger cohort in the original Hawaii Aging with HIV Cohort-Cardiovascular Disease (HAHC-CVD) study [35].

### Higher variability of systemic levels of inflammatory factors is characteristic of insulin resistance

To determine the relationship between IR and IS with respect to inflammation, we measured plasma levels of inflammatory cytokines and chemokines using Luminex-based assays and evaluated the leukocyte cell composition in PBMCs among our cohort. Results of these immunological measures stratified between the IS and IR groups are shown in Table 2. Of the cytokines/chemokines measured, significantly higher levels of MCP-1 was observed in IR compared to IS individuals (*P =* 0.04; Table 2). MCP-1 is an essential factor involved in the migration of monocytes to tissues, which may underlie microinflammation in skeletal muscle and adipose tissue in the IR state [56]. Increased systemic MCP-1 concentrations agrees with previous reports of associations with IR and diabetes [57]. The other cytokines/chemokines (i.e., IL-8, IL-1β, TNF-α, IFN-γ, IL-6, and VEGF) did not elicit significant differences between IR and IS participants. However, we observed higher variability in the levels of all these inflammatory markers in IR compared to IS individuals (Table 2).

We next measured the frequency of total monocytes (CD56,19,20^low^ and HLA-DR^+^), and classical (CD14^++^, CD16^-^), intermediate (CD14^++^, CD16^+^), and non-classical (CD14^+^, CD16^++^) monocyte subtypes in our cohort using immunophenotyping analysis (Additional file 2: Figure S2A). The frequency of total monocytes and monocyte subsets derived from PBMCs of individuals in either the IR or IS groups were equivalent (Table 2; Additional file 2: Figure S2B). However, similar to the plasma levels of inflammatory markers, we observed a considerably higher degree of variability in the abundance of total monocytes in the IR compared to IS group (Table 2).

### Monocyte methylation signature of insulin resistance relates to immune function

As suggested by our previous study, monocytes exhibited a specific methylomic signature of neurocognitive defects in HIV-infeced individuals [32]. To determine whether monocytes also exhibit a methylomic signature of insulin resistance, we first enriched monocytes in our cohort of 37 individuals. We confirmed the purity of our enrichment procedure by flow cytometry-based immunophenoyping, which yielded an average monocyte purity of 89% from all individuals in the IS and IR group (Table 2). Further, we independently verified monocyte composition and purity using a comparative DNA methylomic approach with data we collected using the 450 K microarray. To do so, we first identified a monocyte-specific DNA methylation profile using data available from GEO Accession: GSE35069 [45] and an approach similar as we had previously reported [32]. We then compared the methylation states of these CpGs underlying the monocyte-specific profile with that of each of the enriched populations of monocytes from our cohort. This resulted in a significant positive correlation in the IS and IR groups (*r =* 0.96 and *r* = 0.96, respectively; *P* < 0.0001 for both; Additional file 2: Figure S2C), suggesting that the monocyte-specific methylation states matched that derived from our monocytes we enriched from PBMCs. Additionally, when evaluating DNA methylation states at CpGs that specify the PBMC population in bulk using data from GSE35069 [45], we observed that these loci in the enriched population of monocytes from IS and IR individuals were significantly distinct from the PBMC-specific methylation profile (*r =* 0.27 and *r* = 0.28, respectively; *P* < 0.0001 for both; Additional file 2: Figure S2D), reinforcing the purity of monocytes that we enriched from PBMCs. To further confirm immunophenotyping data, we identified monocyte subset-specific DNA methylation patterns unique to classical, intermediate, and non-classical monocytes and compared these profiles with that derived from total monocytes in IR and IS individuals. By comparing the correlation coefficient derived from comparing the methylation states at CpGs that were subtype-specific for each of the three monocyte subtypes with that of each individual in our cohort, we observed no significant differences between IR and IS individuals among classical monocytes (mean ± SEM; IR: 0.78 ± 0.004, IS: 0.78 ± 0.06; Additional file 2: Figure S2E), intermediate monocytes (IR: 0.77 ± 0.008, IS: 0.76 ± 0.01; Additional file 2: Figure S2F), and non-classical monocytes (IR: 0.63 ± 0.004, IS: 0.63 ± 0.006; Additional file 2: Figure S2G). Altogether, these methylation profiling analyses confirmed immunophenotyping data indicating that IS and IR indviduals exhibited no differences in monocyte cell type and subtype composition.

**Fig. 1:**
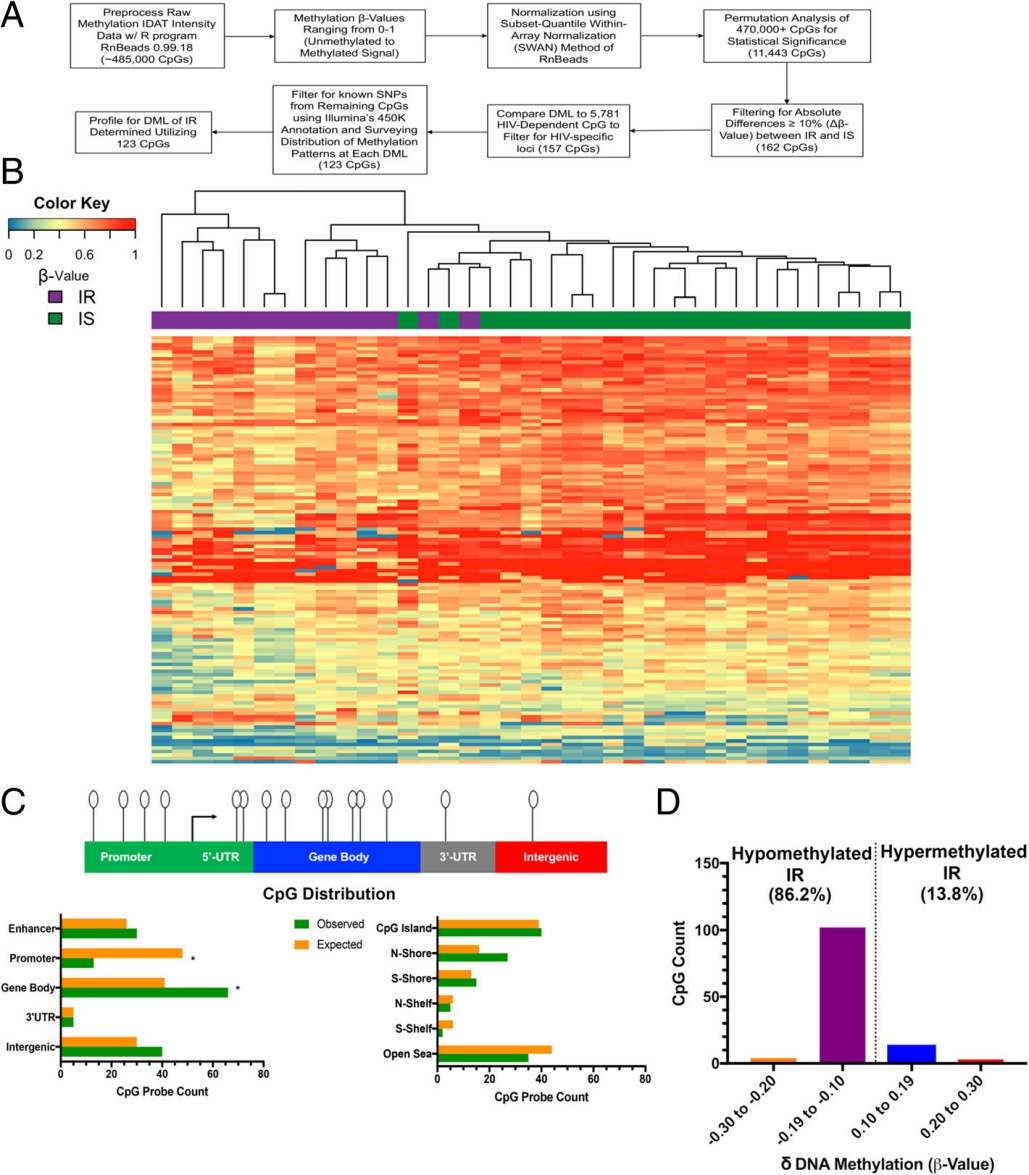
Differentially methylated loci from IS and IR individuals. **a** Workflow for processing of DNA methylation data acquired from 450K microarray to generate DMLs. **b** Heatmap produced from unsupervised hierarchical clustering using the Manhattan distance, complete linkage method displaying DNA methylation (β-value) of the 123 CpGs determined to be DMLs, stratifies IS (green; above column) and IR (purple; above column) from each other. DNA methylation ranges from low (0, blue) to intermediate (0.50, yellow) to high (1.0, red). **c** Distribution of DMLs in the gene context, with representation of CpGs as lollipops distributed linearly along gene regions indicated (top diagram). Distribution of CpGs along the indicated gene region with expected (orange) and observed (green) frequency over the 450K array shown and compared (left graph). Distribution of CpGs relative to position in CpG islands with expected (orange) and observed (green) frequency over the 450K array shown and compared (right graph). *P < 0.05. **d** Histogram representation of the distribution of the *δ* value of IR for all 123 DMLs, as described in methods. Majority of loci were hypomethylated in IR compared to IS

Having confirmed the purity of the monocyte methylation dataset in each of the samples we enriched from PBMCs from all individuals in our cohort, we then identified differentially methylated CpGs between IR and IS individuals. To do so, we applied a permutation analysis of the entire 450 K array to compute the sampling distribution between both groups with a threshold *P ≤* 0*.*05. This yielded 11,443 CpGs that exhibited some degree of differential methylation between the groups. Similar as we previously reported [32], we then filtered this list based on absolute mean differences in methylation of 10% or more between the IR and IS groups (*δ* ≥ |0.10| at *P* < 0.05), which yielded 162 CpGs that we define as differentially methylated loci (DMLs) associated with IR. As all individuals were HIV-infected despite being stratified on the basis of IR, we further confirmed that these DMLs related to IR rather than HIV infection status. To do so, we generated a monocyte methylomic profile related to HIV status by comparing the monocyte methylation states from our cohort to that of HIV-seronegative volunteers (*n* = 9) by the same methods as described above. We performed a permutation analysis to identify DMLs between HIV-seronegative and HIV-infected (*n* = 37) individuals, which resulted in 5,781 CpGs exhibiting absolute mean differences in methylation greater than 10% between these groups (*δ* ≥ |0.10| at *P* < 0.05). By comparing these 5,781 CpGs, potentially HIV-associated DMLs, to the 162 CpGs related to IR in monocytes from HIV-infected individuals, we could discern those CpGs in the IR analysis whose methylation differences may be attributed to HIV infection status. Union of these two lists of DMLs revealed that only 5 CpGs (~ 3%) overlapped. Upon removing these sites, we next filtered out any loci that exhibited known single-nucleotide polymorphisms (SNPs) or displayed a pattern of methylation distribution that could be confounded by the presence of a SNP within the probe [58]. To do so, we manually assessed the distribution of methylation levels of DMLs within either the IR or IS group that exhibited *β* values corresponding to 0%, 50%, or 100% methylation, a pattern which cannot be distinguished from a SNP, and/or annotated by Illumina to possibly harbor a SNP (https://support.illumina.com/downloads/infinium_humanmethylation450_product_files.html). This assessment revealed 33 CpGs among the DMLs related to SNPs, which we removed from further analyses. Altogether, using these stringent filtering criteria, we identified 123 CpGs whose methylation states in monocytes robustly stratified IR and IS according to the clinical parameter we described (Fig. 1a). We consider these CpGs as HIV-independent DMLs relevant to insulin resistance. Unsupervised hierarchical clustering revealed a strong degree to which the methylation states of these DMLs could distinguish between IR and IS individuals (Fig. 1b). Interestingly, the majority of these DMLs were hypomethylated within the IR group compared to the IS group (106 CpGs, 86.2%; Fig. 1d), a finding consistent with previous studies reporting DNA hypomethylation in PBMCs and in the liver and kidneys in diabetic individuals and rats, respectively [59, 60].

Next, we evaluated whether the IR-associated DMLs preferentially occurred in specific genomic contexts. Specifically, our analyses examined whether the methylation differences were over CpG islands, gene promoters, gene bodies, or intergenic regions of the genome. We found that the DMLs were predominantely distributed and enriched within gene bodies (observed: 66, expected: 41; *P* < 0.05; Fig. 1c) and occurred significantly less than expected at gene promoters (observed: 29, expected: 53; *P* < 0.05; Fig. 1c). A closer inspection of CpGs with respect to their distribution in CpG islands, sequences dense in GC content and typically within promoter regions [61], revealed a trending but a statistically non-significant enrichment of DMLs within the north shore, a region within ~ 2 kb region upstream of CpG islands (observed: 27, expected: 16; Fig. 1c). Interestingly, these CpG shores have some of the most dynamic changes in DNA methylation, especially in disease [62]. These data suggest IR-associated differences of DNA methylation that may relate to epigenetic dysregulation of genes involved in monocyte function. To further explore this possibility, we next performed gene ontology (GO) enrichment analysis using Enrichr [46, 47]. This analysis revealed five genes, in particular, that contained DMLs significantly overrepresented in pathways involved in glucose metabolism, inflammation, and other pathways related to insulin sensitivity: *NDUFS7*, *INSIG1*, *RAB1A*, *CMKLR*, and *MAPK11* (Table 3) [63–70]. Additionally, DNA methylation levels of the CpGs in four of these five genes were significantly correlated to HOMA-IR: *MAPK11* (*r* = − 0.42; *P* = 0.009), *NDUFS7* (*r* = − 0.39; *P* = 0.02), *RAB1A* (*r* = − 0.38; *P* = 0.02), and *CMKLR* (*r* = − 0.50; *P* = 0.001 [Additional file 3: Figure S3A–D]). These results implicate a role for DNA methylation in regulating genes involved in monocyte activity, which may be dysregulated as a result of the IR condition.

**Table 3:**
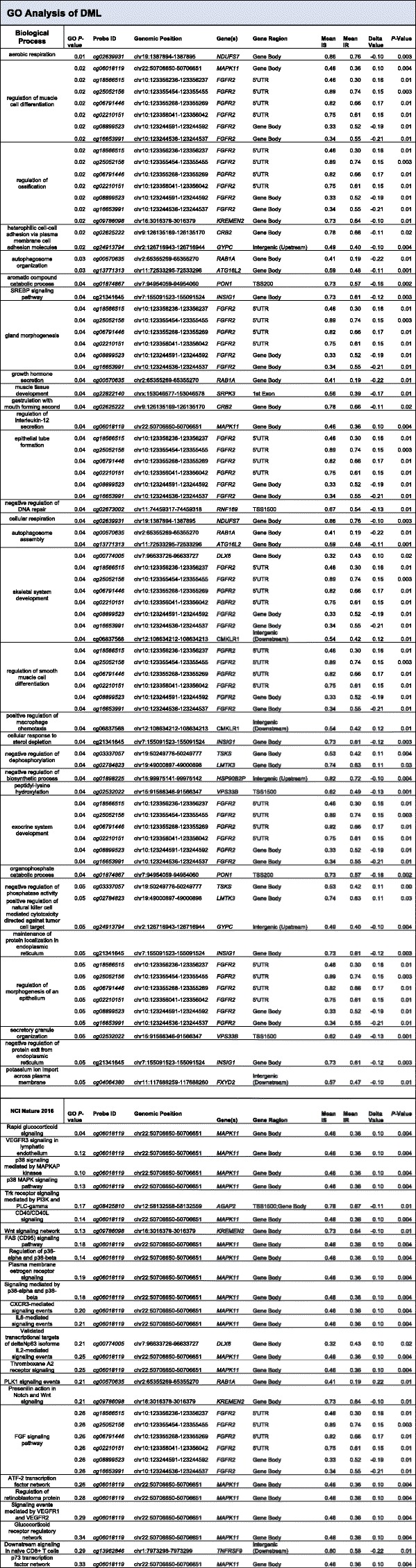
GO Analysis of DMLs

DMLs enriched at genes involved biological processes as indicated with CpG probe ID and genomic position (hg19) displayed. GO *P* value was determined using Fisher’s exact test with significance at *P* < 0.05. Delta (휕) value represents the difference in DNA methylation (*β* value) of the DML between the mean methylation levels of IS and IR groups. *P* value represents the significance of the mean differences between IS and IR calculated by Mann-Whitney *U* test, with significance at *P* < 0.05

*DML* differentially methylated loci, *CpG* cytosine guanine dinucleotide, *UTR* untranslated region, *TSS* transcription start site

We further sought to explore the degree to which the DML associated with IR identified among HIV-infected individuals may be specific to HIV infection status or represented shared gene pathways affected by IR independent of HIV infection. To do so, we took advantage of a small but robust sampling of 450 K array methylation data we gathered from monocytes in 9 HIV-seronegative individuals with differences in HOMA-IR that stratified into IR (*n* = 5; avg. HOMA-IR > 3.0, note 3/5 diagnosed with T2D; Additional file 5: Table S3) and IS (*n* = 4; avg. HOMA-IR: 0.88 [0.73–1.20]; Additional file 5: Table S3) groups. Using an approach similar to that described for the HIV-infected cohort, we performed a permutation analysis on DNA methylation differences in monocytes between HIV-seronegative individuals, which resulted in 3,296 CpGs. We further filtered out CpGs that did not meet an absolute mean difference in methylation greater than 10% between these groups (*δ* ≥ |0.10| at *P* < 0.05), resulting in 345 CpGs with statistically significant and biologically relevant differences in DNA methylation between HIV-seronegative IR and IS individuals; we labeled these CpGs as HIV-seronegative DML of IR. This list was further refined to filter out CpGs at known SNPs and CpGs that displayed methylation patterns indistinguishable of potential unknown SNPs, which resulted in the removal of 41 CpGs (refer to Additional file 4: Figure S4A for workflow). Unsupervised hierarchal clustering of the remaining 304 CpGs distinctly clustered IR and IS individuals into their respective groups, further supporting a methylomic profile in monocytes that could distinguish the IR condition and potential biomarkers for disease risk (Additional file 4: Figure S4B). We observed that among the 304 CpGs in the HIV-seronegative DML of IR, only 1 CpG (< 1%) overlapped with the 123 CpGs characterizing the DML of IR in the HIV-infected cohort (Additional file 4: Figure S4C). However, GO analysis of the 304 CpGs found enrichment at 275 genes that were involved in pathways similar to that of the DML of IR in HIV-infected individuals, including pathways relevant to immune function, metabolism, and signaling pathways (Additional file 6: Table S2). These results indicate that although methylation differences at specific CpGs may be distinct between monocytes from HIV-infected and non-infected individuals, those whose methylation states robustly distinguished individuals on the basis of insulin resistance occur at genes in shared biological pathways relevant to monocyte function, insulin signaling, and glucose metabolism. Additionally, we estimated monocyte subset proportion in monocytes of these HIV-seronegative individuals based on our subset-specific methylation profiles using a method similar to that described for the HIV-infected individuals. Unlike the HIV-infected population (Additional file 2: Figure S2), we observed a slight but significant decrease in the correlation of only the intermediate monocyte subset in HIV-seronegative IR compared to IS individuals (Additional file 4: Figure S4D). Along with differences in monocyte methylation, the effect on monocyte subsets may be important in contributing to the insulin-resistant condition independent of HIV infection. However, we cannot completely rule out the possibility that HIV infection may play a role in differences we observed between IR and IS given limitations of age, sex, and clinical differences among the HIV-seronegative individuals. Thus, we further focused on understanding the differences in methylation among the HIV-infected individuals.

### Methylation levels at distinct CpGs in monocytes strongly associate with insulin resistance, which may originate from aberrant maintenance of an HSC-like state

**Fig. 2:**
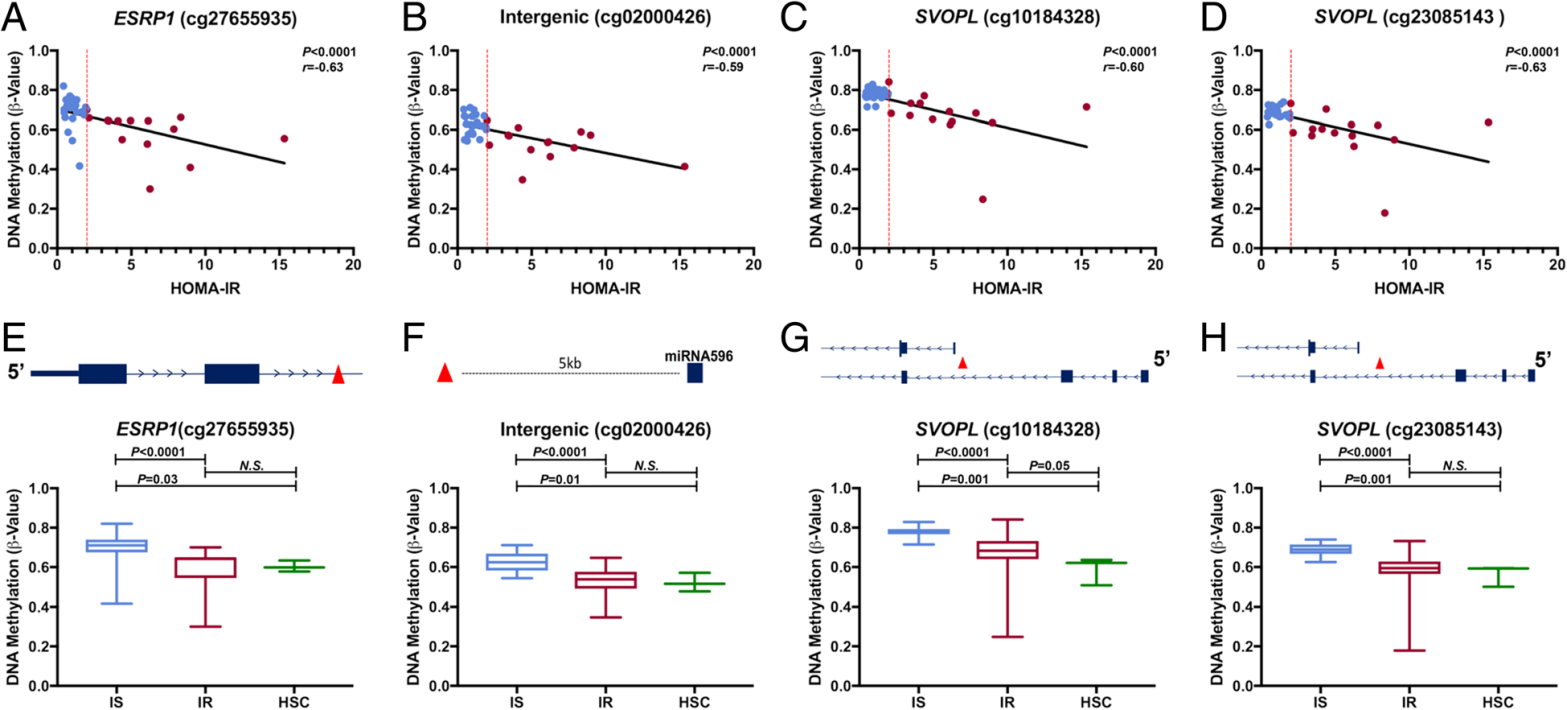
Logistic regression analysis reveals DMLs that may be predictive of insulin sensitivity status and whose methylation states in monocytes of individuals with IR strongly associate with that of HSCs. **a**–**d** Four CpGs were identified as significantly associated with IR outcome using simple logistic regression analysis of the DMLs, clinical data, and immunological data each of which independently predicted outcome. Linear regression analysis was applied to display this relationship between HOMA-IR scores and DNA methylation at each of the four CpGs and their associated genes for **a** ESRP1 (cg27655935), **b** an intergenic region 5kb from miRNA596 (cg02000426), **c** SVOPL (cg10184328), and **d** SVOPL (cg23085143). Blue dots represent data from IS individuals, and red dots represent data from IR individuals. Red dotted line shows the cut-off value for HOMA-IR. Spearmen correlation coefficient (*r*) is shown with significance at *P* < 0.05. **e**–**h**. Box-plots shows the monocyte DNA methylation levels of the four independently predictive CpGs in the IS and IR groups along with the methylation states in HSCs for **e** ESRP1 (cg27655935), **f** an intergenic region 5kb from miRNA596 (cg02000426), **g** SVOPL (cg10184328), and **h** SVOPL (cg23085143). Differences between these groups were determined by Mann-Whitney *U* test with significance at *P* < 0.05. Diagrams above each box-plot shows a linear depiction of the associated gene with the position of each CpG indicated by a red triangle. Blue boxes represent exonic sequences with gene orientation marked (5′- ends). ESRP1, Epithelial Splicing Regulator Protein 1; SVOPL, SV2-Related Protein Homolog-Like; HSC, hematopoietic stem cell

To identify parameters that may be predictive of insulin resistance, we performed multiple logistic regression analyses among the 123 DMLs of IR identified in the HIV-infected cohort combined with clinical data and immunological data. We found that individual methylation levels at any 1 of 4 CpGs had the strongest effect on IR outcome independent of other risk factors: *cg27655935* (*ESRP1*), *cg02000426* (Intergenic), *cg10184328* (*SVOPL*), and *cg23085143* (*SVOPL*). Due to a limited sample size and multicollinearity of some data points, simple logistic regression analyses were the strongest predictive models of outcome, or IR. To examine the degree to which the DNA methylation levels at these CpGs associated with HOMA-IR scores, we performed a correlation analysis. For all four CpGs, we observed a significant inverse relationship between DNA methylation and HOMA-IR scores: a CpG within *ESRP1* (*cg27655935*; *r* = − 0.63; *P* < 0.0001; Fig. 2a), an intergenic CpG ~ 5 kb from miRNA596 (*cg02000426*; *r* = − 0.59; *P* = 0.0001; Fig. 2b), and two CpGs within a putative alternate promoter of *SVOPL* (*cg10184328*: *r* = − 0.60; *P* < 0.0001; Fig. 2c and *cg23085143*: *r* = − 0.63; *P* < 0.0001; Fig. 2d). As a result of our simple logistic regression analyses, we determined the methylation level (*β* value) for each CpG that predicted insulin resistance independently of the other three CpGs or risk factors. In each model, a *β* value less than 0.61, 0.73, 0.64, or 0.56 for *ESRP1* (*cg27655935*), *SVOPL* (*cg10184328*), *SVOPL* (*cg23085143*), or the intergenic CpG (*cg02000426*), respectively, classified IR with 64.3% accuracy and values greater than or equal to these cutoffs classified IS individuals with 90.2% accuracy (data not shown). To compare the predictive value of the methylation level at these sites with that of the clinical risk factors typically associated with IR, we performed several multiple logistic regression analyses on clinical and immunological data independent of CpG methylation, which generated four independent logistic regression models that each significantly associated with IR (Table 4). As measured by the area under the curve (AUC), we observed a stronger diagnostic accuracy of predicting IR by simple logistic regression models based on methylation states at any of the four CpGs compared with models based on any of the clinical risk factors (Table 4). Our data suggest DNA methylation at specific loci in monocytes may have potential value as epigenetic biomarkers predictive of IR.

**Table 4:**
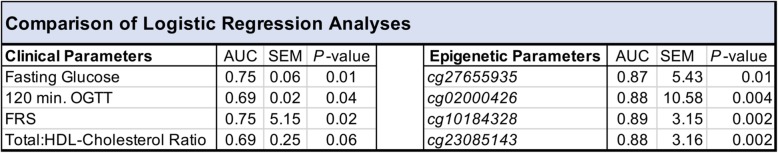
Multiple independent logistic regression analyses of clinical/immunological data and clinical/immunological/methylation data reveal the latter produces stronger probability of predicting IR

Separate logistic regression analyses were performed for clinical and immunological data (left panel) and a combination of clinical, immunological and epigenetic data (right panel). The strongest independent predictive models (AUC) for outcome (IR) for clinical/immunological data were either, fasting glucose, 120 min. OGTT, FRS, or total to HDL cholesterol ratio. The strongest independent predictors of IR for clinical/immunological/epigenetic data were the methylation states of four CpGs; cg27655935, cg02000426, cg10184328, or cg23085143. Significance at *P* < 0.05

*AUC* area under the curve, *OGTT* oral glucose tolerance test

To further explore the possibility that the methylation levels at the four CpGs may relate to the development of IR [71], we next determined how the levels of methylation at these sites in monocytes compared to that from an earlier timepoint in myeloid differentiation, specifically to hematopoietic stem cells (HSCs) from which monocytes are derived. Thus, using a publicly available 450 K array dataset of human HSCs from cord blood cells (*n* = 3; GEO Accession GSE87197) [44] and normalized using the same approach as we have described for monocytes in our study, we performed a comparative analysis of the average methylation levels at these sites in HSCs with that of monocytes from the IS and IR group. For *cg2765593* in *ESRP1*, we observed a significantly higher level of methylation in the IS group than that in the IR group (mean ± SEM; IS: 0.69 ± 0.02, IR: 0.59 ± 0.03; *P* < 0.0001; Fig. 2e). Interestingly, the levels of methylation at this site in HSCs were similar to that of monocytes from IR individuals (HSC: 0.60 ± 0.02, IR: 0.59 ± 0.03; Fig. 2e), which was distinct from IS individuals (HSC: 0.60 ± 0.02, IS: 0.69 ± 0.02; *P =* 0.03; Fig. 2e). Notably, *ESRP1* has previously been shown to regulate differentiation of stomach smooth muscle cells and is involved in regulation of alternative splicing of *FGFR2*, a gene that regulates the differentiation of HSCs [72–74]. For *cg02000426*, a CpG in an intergenic region of chromosome 8 approximately 5 kb from *miRNA596*, we observed significantly higher levels of methylation in the IS compared to IR group (IS: 0.63 ± 0.01, IR: 0.53 ± 0.02; *P <* 0.0001; Fig. 2f). Similar to *ESRP1*, the methylation level at this CpG in HSCs was indistinguishable from that of monocytes of IR individuals (HSC: 0.52 ± 0.03, IR: 0.53 ± 0.02; Fig. 2f), while it remained significantly different from that of IS individuals (HSC: 0.52 ± 0.03, IS: 0.63 ± 0.01; *P* = 0.005; Fig. 2f). Although the role *miRNA596* has not been completely elucidated, some studies have observed microRNA involvement in HSC function [75]. For cg10184328 and *cg23085143*, two CpGs within 200 bp of each other within *SVOPL*, exhibited higher levels in methylation in the IS group compared to that in the IR group (IS: 0.78 ± 0.01, IR: 0.67 ± 0.04; *P <* 0.0001 and IS: 0.69 ± 0.01, IR: 0.58 ± 0.03, *P < 0.0001*, respectively; Fig. 2g, h). When compared to the methylation levels in HSCs, both CpGs exhibited methylation levels indistinguishable in monocytes from IR individuals (HSC: 0.59 ± 0.04, IR: 0.67 ± 0.04 and HSC: 0.56 ± 0.03, IR: 0.58 ± 0.03, respectively; Fig. 2g, h), yet remained distinct from that of IS individuals (HSC: 0.59 ± 0.04, IS: 0.78 ± 0.01, *P* = 0.001, and HSC: 0.56 ± 0.03, IS: 0.69 ± 0.01, *P* = 0.001, respectively; Fig. 2g, h). Although limited, one study has shown that *SVOPL* was involved in HSC function [76]. Altogether, the methylation levels in monocytes at all four of these CpGs that strongly associate with IR appear to be similar to that established early in HSCs, hinting at a potential source of the IR-associated methylation levels in monocytes.

**Fig. 3:**
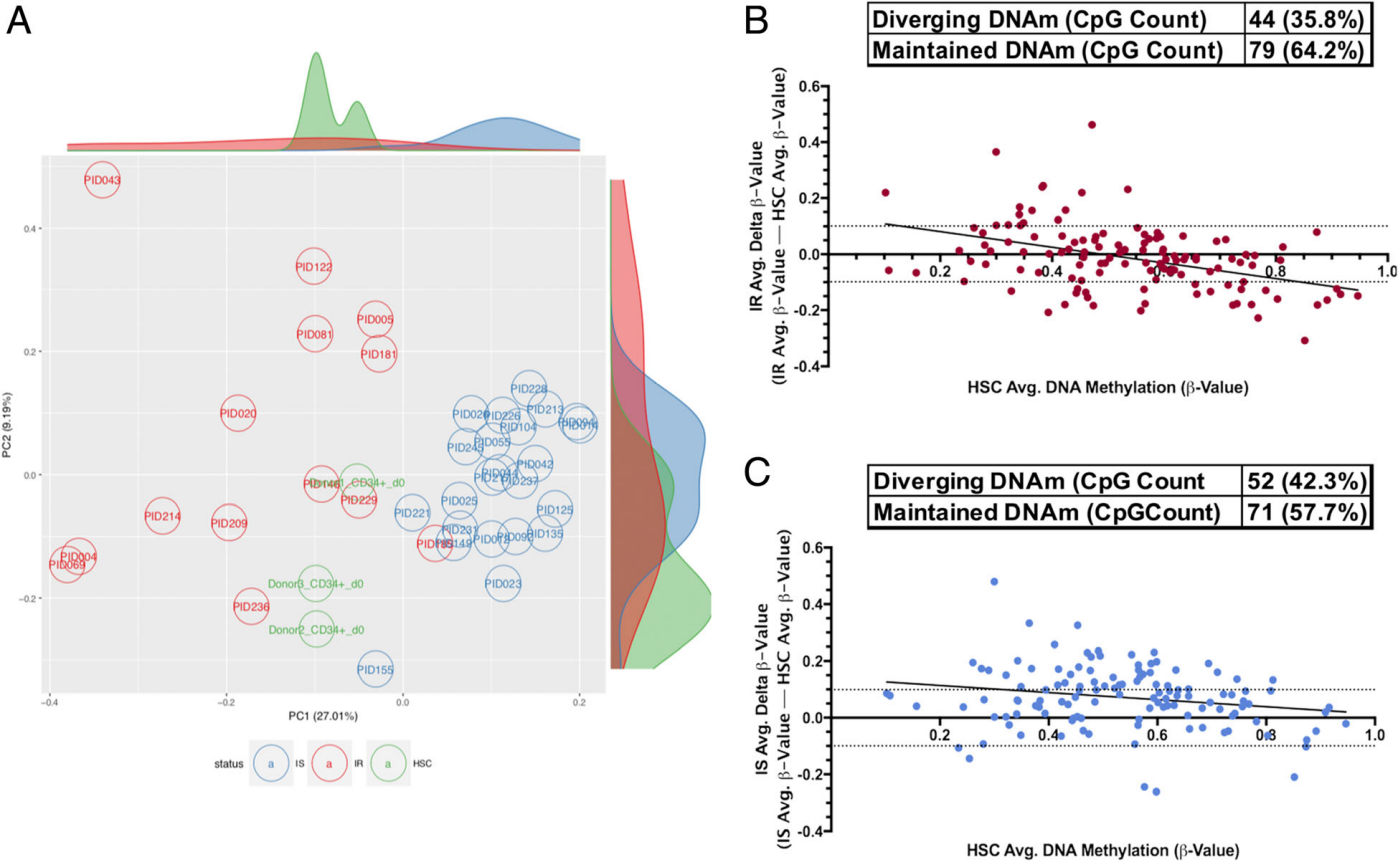
Monocyte methylation levels of DMLs vary considerably more in IR individuals compared to that of IS and maintain an HSC-like state. **a** Scatter plot of the first two principal components displays the distribution of variability of the DNA methylation at the DMLs for monocytes of IS (blue) and IR (red) individuals, with methylation data at these sites from HSCs (green) shown. Peaks distributed on the *X* axis (upper perimeter) and *Y* axis (right perimeter) represent the density of samples present on that axis with matched colors. **b** and **c** Representation of the degree of divergence and/or maintenance of methylation between IR (**b**) or IS (**c**) and HSCs of each DML. Dotted line represents cut-off (10% difference in methylation of the *δ* value) for divergence from HSCs. Values ≤ − 0.10 represents hypomethylation in either the IR or IS group vs HSCs, whereas values ≥ 0.10 represents hypermethylation in either the IR or IS group vs HSCs. Values ≤ 0.10 but ≥ − 0.10 are loci considered to maintain methylation levels in either the IR or IS group to a similar degree as in HSCs. Table above **b** and **c** shows the count and frequency (%) of DMLs that diverge or maintain methylation levels relative to HSCs

Given the origin of circulating monocytes from HSCs, the results described above suggest the intriguing possibility that methylation states observed in monocytes under insulin-resistant conditions may arise as a result of maintaining an HSC-like methylation state during myeloid differentiation and thus a failure to adopt a monocyte-specific state in IR individuals. To determine whether this may be the case, we compared the methylation levels of all IR-associated DMLs to that of HSCs. To do so, we applied a principal component analysis (PCA) using the methylation states of the 123 DMLs in monocytes from IS and IR individuals as well as that of HSCs. Examining the first two principal components derived from this dataset (explaining ~ 27% and ~ 9% of the variance in the data, respectively) revealed that in comparison to that of IS individuals, the methylation levels from monocytes of IR individuals tended to be more variable and related more to that of HSCs (Fig. 3a). To determine which CpGs may be driving the IR-association with HSCs, we first subtracted the normalized *β* values of monocytes in both IR and IS groups from that of HSCs. Although we observed slightly more CpGs among the IR group whose methylation levels were indistinguishable from that of HSCs (79 vs 71 CpGs; Fig. 3b, c), they overlapped on 35 CpGs. GO analysis of the 79 CpGs whose methylation states in IR individuals were similar to that of HSCs were enriched at genes involved in pathways relevant to regulating pluripotency of stem cells (*FGFR2*), Lck and Fyn tyrosine kinases in initiation of T cell receptor activation (*CD3E*), and Wnt signaling (*PCDHGB7/PCDHB11* and *SMARCB1*; Table 5). In contrast, GO analysis of the 71 CpGs whose methylation states in IS individuals were similar to that of HSCs were enriched at genes involved in T cell proliferation (*MCI1* and *TNFRSF9*; Table 5). Interestingly, these results suggest possible CpG sites whose methylation states must be maintained throughout myeloid differentiation and may be necessary for proper monocyte function in the IS condition. Notably, these data also reveal CpG sites whose methylation states, if aberrantly maintained in an HSC-like state, potentially contributes to abnormal monocyte differentiation and function that associates with the higher degree of variability in systemic inflammation and clinical factors associated with insulin resistance. We caution, however, that this analysis was performed on a limited set of individual HSCs (*n* = 3) from which 450 k array data was publicly available. Future studies are required to validate these preliminary observations.

**Table 5:**
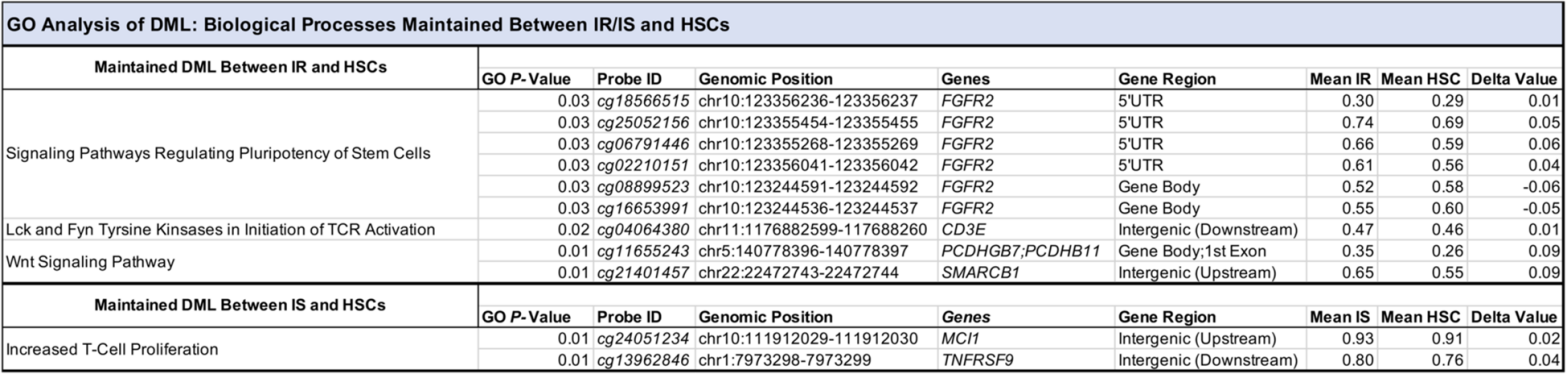
GO analysis of DML maintaining DNA methylation between HSCs and IR/IS subjects

GO analysis of CpGs that maintained DNA methylation between HSCs and IR or IS individuals. Fisher Exact Test was used to determine significance of gene enrichment in biological processes with significance at *P* < 0.05. Delta (휕) value was derived from difference of DNA methylation levels (β-value) between IR or IS and HSCs at specific DMLs. Probe ID (CpG), associated gene, and gene position (hg19) are indicated

## Discussion

Inflammation has become a prominent feature studied in the pathogenesis of insulin resistance, T2D, and CVD [12]. Monocytes play a crucial role in innate immunity, and their activation is important to the immune response in fighting infection or clearing dead cells and debris, but the inability of these cells to resolve inflammation appears to underlie the pathogenesis of cardiometabolic disorders [77, 78]. MCP-1, primarily released by monocytes, macrophages, and dendritic cells, is a key chemokine involved in trafficking of monocytes to targeted tissue to elicit local inflammation and has previously been associated with IR and T2D [57, 79]. Despite the HIV infection status of all individuals in our cohort, we observed elevated systemic levels of MCP-1 among those who were insulin resistant (defined by HOMA-IR; Table 2), which could be indicative of their heightened inflammatory states and increased risk of developing cardiometabolic disease. Indeed, insulin-resistant individuals also exhibited a higher FRS score compared to insulin-sensitive individuals (Table 1). Based on a previous study describing increased abundance of total monocytes, including the classical subset (CD14^++^CD16^-^) among insulin-resistant HIV-infected individuals which predicted HOMA-IR [80], we initially suspected that the differing levels of MCP-1 in our cohort may relate to variability of the monocyte population. However, our flow cytometry-based data of monocyte frequencies, including the three characterized monocyte subsets, showed no differences among insulin-sensitive and insulin-resistant individuals (Table 2; Additional file 2: Figure S2B). We further confirmed this by utilizing monocyte-specific and monocyte subset-specific DNA methylation data (Additional file 2: Figure S2C, E, F). Thus, among our cohort at least, non-monocyte sources of MCP-1 (i.e., macrophages and dendritic cells) are suspected to contribute to the variability between the two groups. Additionally, our data suggest that differences in monocyte function, rather than the proportion and composition, may exist between insulin-sensitive and insulin-resistant individuals.

Given the role of DNA methylation in regulating cell identity and function [81–83], we examined the methylomic landscape of monocytes in insulin-sensitive and insulin-resistant individuals. Our comparative analyses revealed 123 DMLS whose methylation states robustly stratified insulin-resistant and insulin-sensitive individuals as illustrated by the near complete clustering (Fig. 1b), similar to our previous work describing DNA methylation differences in monocytes between HIV-infected individuals with and without cognitive impairment [32]. Similar associations stratified insulin-resistant and insulin-sensitive individuals with DNA methylation differences in visceral adipose tissue [84]. These differences occurred preferentially downstream of promoter regions, in particular within gene bodies and CpG shores (Fig. 1c). Notably, these regions contain the highest DNA methylation dynamics, often exhibiting tissue/cell type specificity [62]. Methylation in these transcribed regions could be relevant to regulating gene expression via alternate promoter usage or alternative splicing [85]. Interestingly, we observed DNA hypomethylation in the DMLs of insulin-resistant individuals (Fig. 1d), a finding similar to a previous report describing extensive DNA hypomethylation in pancreatic beta islet cells from patients with T2D [86]. Hypomethylation may underlie variable monocyte function between insulin-resistant and insulin-sensitive individuals by impacting gene regulation. To explore this possibility, our gene ontology analysis (Table 3) revealed that most of the DMLs we observed that relate to insulin resistance occured at genes involved in aerobic respiration (*NDUFS7*), growth hormone secretion (*RAB1A*), positive regulation of macrophage chemotaxis (*CMKLR1*), and rapid glucocorticoid signaling (*MAPK11*). Functionally, these genes have all been previously described in pathways relating to insulin resistance via signaling or inflammatory pathways [63–70]. Furthermore, each of the CpGs annotated to these genes occurred in intragenic regions and exhibited DNA methylation levels that were significantly inversely correlated with insulin resistance (Additional file 3: Figure S3A–D), further implicating a functional role for the methylation in monocyte activity related to the pathogenesis of insulin resistance.

In addition to implicating gene pathways that may be epigenetically dysregulated in insulin resistance, we sought to explore the feasibility of DMLs from monocyte in predicting the insulin-resistant state, as previous models utilizing DNA methylation in the bone marrow and PBMCs have reported [87]. Our multiple logistic regression analysis identified four CpGs whose methylation states independently associated with HOMA-IR status, which was surprisingly stronger than that of clinical measures typically associated with insulin resistance (Table 4). This finding agreed with prior studies incorporating DNA methylation measures as predictive to health outcome [88]. Interestingly, three of the four CpGs were enriched at genes involved in stem cell pluripotency and differentiation (*ESRP1* and *SVOPL*) [73, 76, 89]. This prompted us to examine how the methylation states at these sites related to an earlier timepoint in myeloid differentiation, in particular as early as HSCs. Our results clearly showed that the methylation states of all four CpGs in monocytes from insulin-resistant, but not insulin-sensitive, individuals significantly resembled the levels established in HSCs (Fig. 2e–h), hinting at a possible origin of DNA methylation in monocytes from insulin-resistant individuals. This notion was further supported by the PCA analysis of all DMLs associated with insulin resistance (Fig. 3a), which also illustrated the heightened variability of DNA methylation levels at these sites among monocytes of IR individuals. These results are consistent with the prior observation of high locus-specific DNA methylation variability in disease states [90, 91].

Variability of DNA methylation, particularly at dynamically regulated sites, such as in gene bodies as we observe here, is a prominant feature of differentiation wherein the chromatin landscape is vulnerable to drastic change. The chromatin landscape of hematopoietic stem cells appears to be less well defined compared to later stages of differentiation [92]. During differentiation, the chromatin landscape adopts a more uniform pattern indicative of their differentiated and more restricted cell fate. However, a recent study examining individual cells in defined populations revealed remarkable diversity in the chromatin landscape of distinct cell types previously characterized on the basis of cell type-specific surface markers [92]. This heterogeneity suggests that among populations of cells thought to be “homogenous,” there could exist multiple, previously uncharacterized subtypes, possibly with distinct functional diversity as well. Given our data showing the higher degree of variability in methylation levels of the monocyte population we described based on the expressivity of monocyte-specific cell surface markers CD14 and CD16 among insulin-resistant and insulin-sensitive individuals coupled with the observation that the methylation levels at these same sites in insulin-resistant (and not insulin-sensitive) individuals appear to be similar to those of HSCs, the monocytes in the insulin-resistant state could be more functionally diverse than their insulin-sensitive counterpart. This diversity in function could potentially explain the significantly higher degree of variability that the IR group exhibits in systemic inflammation and other clinical parameters (Table 2). This notion is further supported by the observation that the CpGs whose methylation states in monocytes of insulin-resistant individuals that were indistinguishable from that of HSCs were overrepresented at genes involved in pathways important to HSC differentiation and pluripotency [93, 94]. Consistent with these findings, increased variability in DNA methylation in immune cells (T cells, B cells, and monocytes) was observed among twins discordant for type 1 diabetes mellitus (T1D), wherein higher variability was observed in the T1D twin as compared to the healthy twin [91]. Furthermore, analysis of cord blood derived from newborns progressing to T1D suggests the differentially variable DNA methylation states may originate from birth, supporting our notion that dysregulation of DNA methylation underlying monocyte function may originate during differentiation.

Taken together, the plasticity of the methylome of monocytes under the insulin-resistant condition may be contributing to the variability of systemic levels of inflammation underlying insulin resistance. Our data implicate the possibility of previously unrecognized monocyte subtypes developing under the insulin-resistant condition, likely due to aberrant myeloid differentiation, that could be contributing to disease progression. Future work characterizing this cell population at the single-cell level could confirm this notion, which would reveal further insights into monocyte heterogeneity and their various functions in insulin resistance.

## Conclusion

Evidence is emerging for what role monocytes, and their tissue differentiated macrophages, may play in the pathogenesis of insulin resistance [29, 80, 95]. Here, we provide the first evidence for a distinct methylomic signature in peripheral monocytes associated with insulin resistance in HIV-infected individuals. The IR-associated DMLs underlying this signature were overrepresented at genes involved in immune function. Further, these DMLs were preferentially distributed at CpG island shores and gene bodies, areas of the genome that are particularly susceptible to epigenetic regulation [62]. Of note, most of these DMLs appeared to be independent of HIV infection status and the methylation states of four of these CpGs strongly accounted for the insulin-resistant condition independent of other clinical risk factors. Together, these results suggest immunoepigenetic biomarkers in monocytes that may be predictive of insulin resistance. Further, our comparison with hematopoietic stem cells suggests that the methylation states of some of these CpGs in monocytes of IR individuals might originate from a failure to undergo normal epigenetic regulation during myeloid differentation. These sites may, for example, be protected from the dynamic changes in methylation during differentiation and consequently alter the trajectory of expression states of underlying genes that impact monocyte function in the insulin-resistant condition. Future research will need to examine whether methylation differences in insulin resistance occur prior to its onset and are functionally relevant, which collectively will lead to improved early diagnostic and treatment strategies that reduce the development of insulin resistance and related chronic conditions in populations with a heightened risk, including HIV-infected and aging populations.

## Additional files

Additional file 1: **Figure S1.** Association between clinical parameters and insulin resistance. A–D. Linear regression analysis of each clinical feature as indicated with insulin resistance (HOMA-IR). Blue dots, insulin-sensitive (IS) individuals; red dots, insulin-resistant (IR) individuals. Dotted red line shows the cut-off for IR and IS groups based on HOMA-IR. Correlation coefficients shown were calculated using Spearman’s rho (*r*), with significance at *P* < 0.05. HOMA-IR, Homeostatic Model Assessment of Insulin Resistance; HDL, high-density lipoprotein cholesterol; BMI, body mass index; FRS, Framingham Risk Score. (PDF 9233 kb)

Additional file 2: **Figure S2.** Independent confirmation of monocyte and monocyte subset composition by flow cytometry and DNA methylation analysis. A. Representative FACS analysis of gating strategy employed for determining monocyte subsets: M1(CD14++,CD16−), M2 (CD14++CD16+), and M3 (CD14+CD16++) monocytes. B. Frequency (%) of monocyte subsets was determined by cellular FACS-based phenotyping of monocytes from IS and IR individuals for classical (M1), intermediate (M2), and non-classical monocytes (M3); significance at *P* < 0.05 by Mann-Whitney *U* test. N.S., non-significant. C. Linear regression analysis validates monocyte enrichment observed by flow cytometry using monocyte-specific DNA methylation data of FACS-sorted cells in comparison to our monocyte enrichment results from IS (blue) and IR (red) individuals as described in the methods section. D. Linear regression analysis of PBMC-specific DNA methylation profiles compared with monocytes enriched from IS (blue) and IR (red) individuals. Significance at *P* < 0.05. Spearman’s rho (*r*) was used to determine correlation coefficients. E–G. Correlation analysis of monocyte subset-specific DNA methylation profiles among IS and IR individuals. Spearman correlation coefficient values (r) were compared between each group for mean differences for indicated monocyte subsets (E: M1, F: M2, and G: M3); Significance at *P* < 0.05. (PDF 9233 kb)

Additional file 3: **Figure S3.** Relationship between DNA methylation and HOMA-IR scores of DMLs enriched at genes involved in various insulin-related processes. DNA methylation derived from DMLs with their CpG probe ID shown that were enriched at genes involved in processes related to insulin signaling: MAPK11 (A), NSUFS7 (B), RAB1A (C), and CMKLR (D). The metylation levels at these CpGs in monocytes were significantly correlated with insulin resistance measured by HOMA-IR from IS (blue dots) and IR (red dots) individuals. Spearman correlation coefficient (*r*) shown with significance at *P* < 0.05. (PDF 9233 kb)

Additional file 4: **Figure S4.** Differentially methylated loci from HIV-seronegative individuals stratified by HOMA-IR into IR or IS. A. Workflow for processing DNA methylation data acquired from 450k microarray to generate the HIV-seronegative DML of IR. B. Unsupervised hierarchal clustering heatmap using Manhattan distance, complete linkage method displays DNA methylation (*β* value) of the 304 CpGs of the HIV-seronegative DML of IR, stratifies IS individuals (green) from IR individuals (purple). DNA methylation ranges from low (0, blue) to intermediate (.50, yellow), to high (1.0, red). C. Vendiagram is representative of the two datasets that culminate the DML of IR in HIV-infected individuals (left; DML of IR) and HIV-seronegative individuals (right; HIV-seronegative DML of IR). Shown are the CpG counts that are independent to each dataset and the CpGs that are overlapped in both datasets; below displays the number of genes harbored at the CpGs. D. Comparison of monocyte subset-specific DNA methylation correlation of HIV-seronegative IR and IS individuals. Correlation determined utilizing Spearman’s rho (*r*); significance determined using Mann-Whitney *U* test between IS and IR at *P* < 0.05. (PDF 9233 kb)

Additional file 5: **Table S3.** Clinical characteristics of HIV-seronegative individuals stratified by insulin sensitivity and insulin resistance. Table represents clinical data comparing the IS and IR groups of HIV-seronegative individuals. Data shown are median values [first quartile, third quartile]. *P* value determined between IS and IR groups using Mann-Whitney *U* test; significance at *P* < 0.05. (PDF 9233 kb)

Additional file 6: **Table S2.** GO analysis of HIV-seronegative DML of IR. Table displays significant biological functions of the DMLs enriched at genes involved in immune responses, metabolic processes, and signaling pathways. Included in table are GO analysis *P* value calculated using the Fisher exact test (*P* < 0.05), the CpG probe ID, genomic position of the DML, the gene(s) harboring each CpG, the genomic location of the CpG in the context of a gene, the mean DNA methylation in both the HIV-seronegative IS and IR groups, the difference in methylation (Delta Value), and the *P* value calculated between the IS and IR groups as determined using the Mann-Whitney *U* test and taken at a significance of *P* < 0.05. (PDF 9233 kb)

Additional file 7: **Table S1.** Datasets used for DNA methylation analyses in this study. List of the GEO accession numbers for the datasets used for DNA methylation analyses incorporated into study. (PDF 9233 kb)

## Data Availability

The datasets used in this study are available in the National Center for Biotechnology Information (NCBI) website’s Gene Expression Omnibus (GEO) at https://www.ncbi.nlm.nih.gov/geo/browse/corresponding to the following GEO Accession number: GSE131627.

## Abbreviations

450 K: Illumina Infinium HumanMethylation450 BeadChip
BMI: Body mass index
CMKLR1: Chemerin chemokine-like receptor 1
CRP: C-reactive protein
DML: Differentially methylated loci
ESRP1: Epithelial splicing regulator protein 1
FACS: Fluorescence-activated cell sorting
FGFR2: Fibroblast growth factor receptor 2
FRS: Framingham Risk Score
GO: Gene Ontology
HAHC-CVD: Hawaii Aging with HIV Cohort-Cardiovascular Disease
HDL-c: High-density lipoprotein cholesterol
HICFA: Hawaii Center for AIDS
HOMA-IR: Homeostatic model assessment of insulin resistance
HSC: Hematopoietic stem cell
IFN: Interferon
IFN-γ: Interferon gamma
IKKβ: I-Kappa-B-Kinase-Beta
IL-10: Interleukin-10
IL-1β: Interleukin-1 beta
IL-6: Interleukin-6
IL-8: Interleukin-8
INSIG1: Insulin-induced gene 1
IR: Insulin resistant
IS: Insulin sensitive
JNK: c-jun N-terminal kinase
LDL-c: Low-density lipoprotein cholesterol
LPS: Lipopolysaccharide
MAP K11: Mitogen-activated protein kinase 11
MCP-1/CCL2: Monocyte chemoattractant protein-1
NDUFS7: NADH:Ubiquinone Oxidoreductase Core Subunit S7
NF-κB: Nuclear factor kappa-light-chain-enhancer of activated B cells
OGTT: Oral glucose tolerance test
PBMC: Peripheral blood mononuclear cell
RAB1A: RAB1A, Member RAS Oncogene Family
SNP: Single-nucleotide polymorphism
SVOPL: SV2-related protein homolog-like
SWAN: Subset-quantile within array normalization
T2D: Diabetes mellitus type 2
TLR: Toll-like receptor
TNF-α: Tumor necrosis factor alpha
VEGF: Vascular endothelial growth factor
