# Supplementary material for: Comparative DNA methylomic analyses reveal potential origins of novel epigenetic biomarkers of insulin resistance in monocytes from virally suppressed HIV-infected adults
Source: Clin Epigenetics. 2019 Jun 28;11:95. doi: 10.1186/s13148-019-0694-1 (PMC6599380; doi:10.1186/s13148-019-0694-1)
Supplement: Supplementary file 1 — Figure S1. Association between clinical parameters and insulin resistance. A–D. Linear regression analysis of each clinical feature as indicated with insulin resistance (HOMA-IR). Blue dots, insulin-sensitive (IS) individuals; red dots, insulin-resistant (IR) individuals. Dotted red line shows the cut-off for IR and IS groups based on HOMA-IR. Correlation coefficients shown were calculated using Spearman’s rho (r), with significance at P < 0.05. HOMA-IR, Homeostatic Model Assessment of Insulin Resistance; HDL, high-density lipoprotein cholesterol; BMI, body mass index; FRS, Framingham Risk Score. (PDF 9233 kb) [file 13148_2019_694_MOESM1_ESM.pdf]

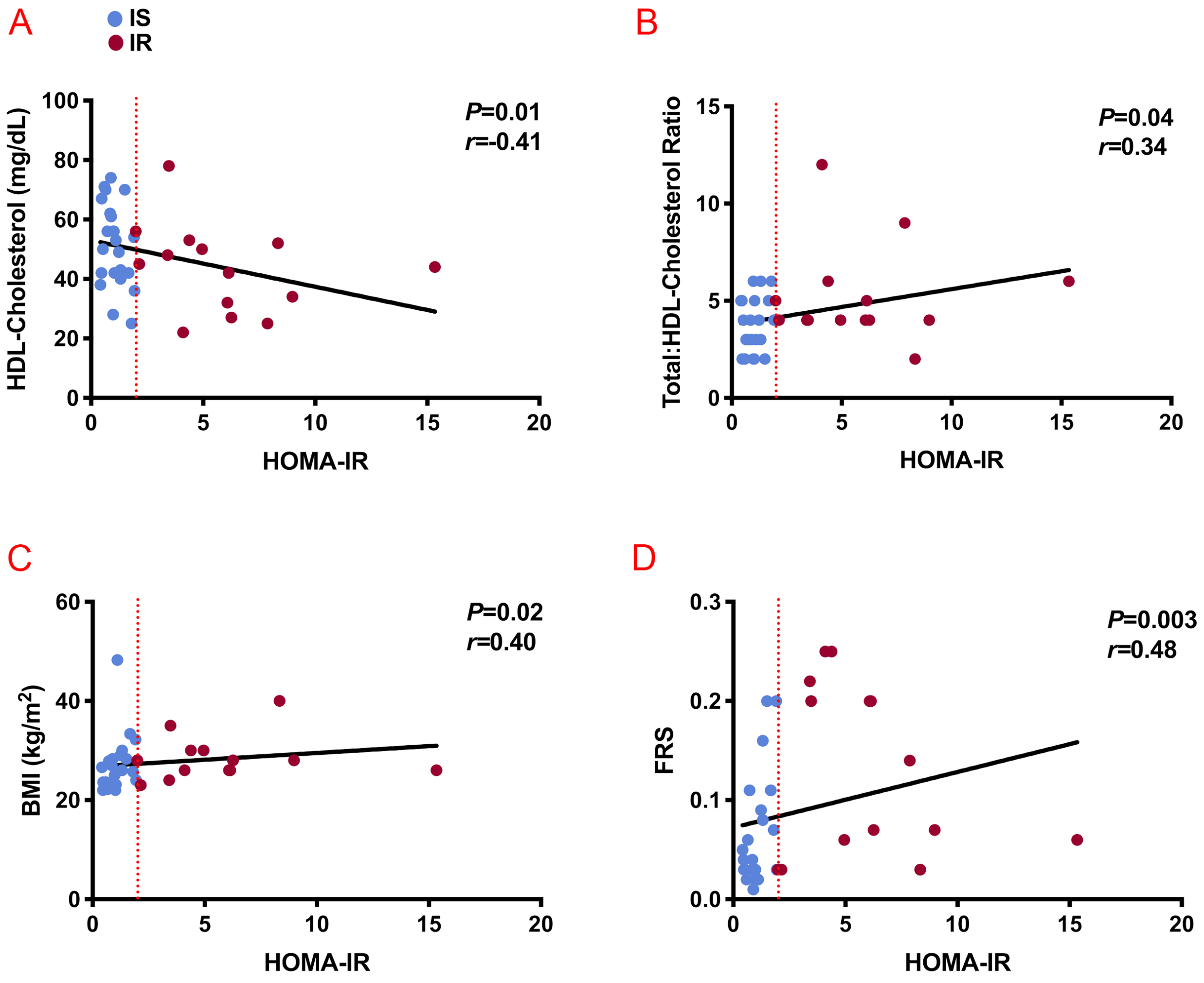

Supplemental Figure 1. Association between clinical parameters and insulin resistance. A-D. Linear regression analysis of each clinical feature as indicated with insulin resistance (HOMA-IR). Blue dots, insulin sensitive (IS) individuals; red dots, insulin resistant (IR) individuals. Dotted red line shows the cut-off for IR and IS groups based on HOMA-IR. Correlation coefficients shown was calculated using Spearman's rho (*r*), with significance at *P*<0.05. HOMA-IR: Homeostatic Model Assessment of Insulin Resistance; HDL: high-density lipoprotein-cholesterol; BMI: body-mass index; FRS: Framingham Risk Score
