# Supplementary material for: Comparative DNA methylomic analyses reveal potential origins of novel epigenetic biomarkers of insulin resistance in monocytes from virally suppressed HIV-infected adults
Source: Clin Epigenetics. 2019 Jun 28;11:95. doi: 10.1186/s13148-019-0694-1 (PMC6599380; doi:10.1186/s13148-019-0694-1)
Supplement: Supplementary file 2 — Figure S2. Independent confirmation of monocyte and monocyte subset composition by flow cytometry and DNA methylation analysis. A. Representative FACS analysis of gating strategy employed for determining monocyte subsets: M1(CD14++,CD16−), M2 (CD14++CD16+), and M3 (CD14+CD16++) monocytes. B. Frequency (%) of monocyte subsets was determined by cellular FACS-based phenotyping of monocytes from IS and IR individuals for classical (M1), intermediate (M2), and non-classical monocytes (M3); significance at P < 0.05 by Mann-Whitney U test. N.S., non-significant. C. Linear regression analysis validates monocyte enrichment observed by flow cytometry using monocyte-specific DNA methylation data of FACS-sorted cells in comparison to our monocyte enrichment results from IS (blue) and IR (red) individuals as described in the methods section. D. Linear regression analysis of PBMC-specific DNA methylation profiles compared with monocytes enriched from IS (blue) and IR (red) individuals. Significance at P < 0.05. Spearman’s rho (r) was used to determine correlation coefficients. E–G. Correlation analysis of monocyte subset-specific DNA methylation profiles among IS and IR individuals. Spearman correlation coefficient values (r) were compared between each group for mean differences for indicated monocyte subsets (E: M1, F: M2, and G: M3); Significance at P < 0.05. (PDF 9233 kb) [file 13148_2019_694_MOESM2_ESM.pdf]

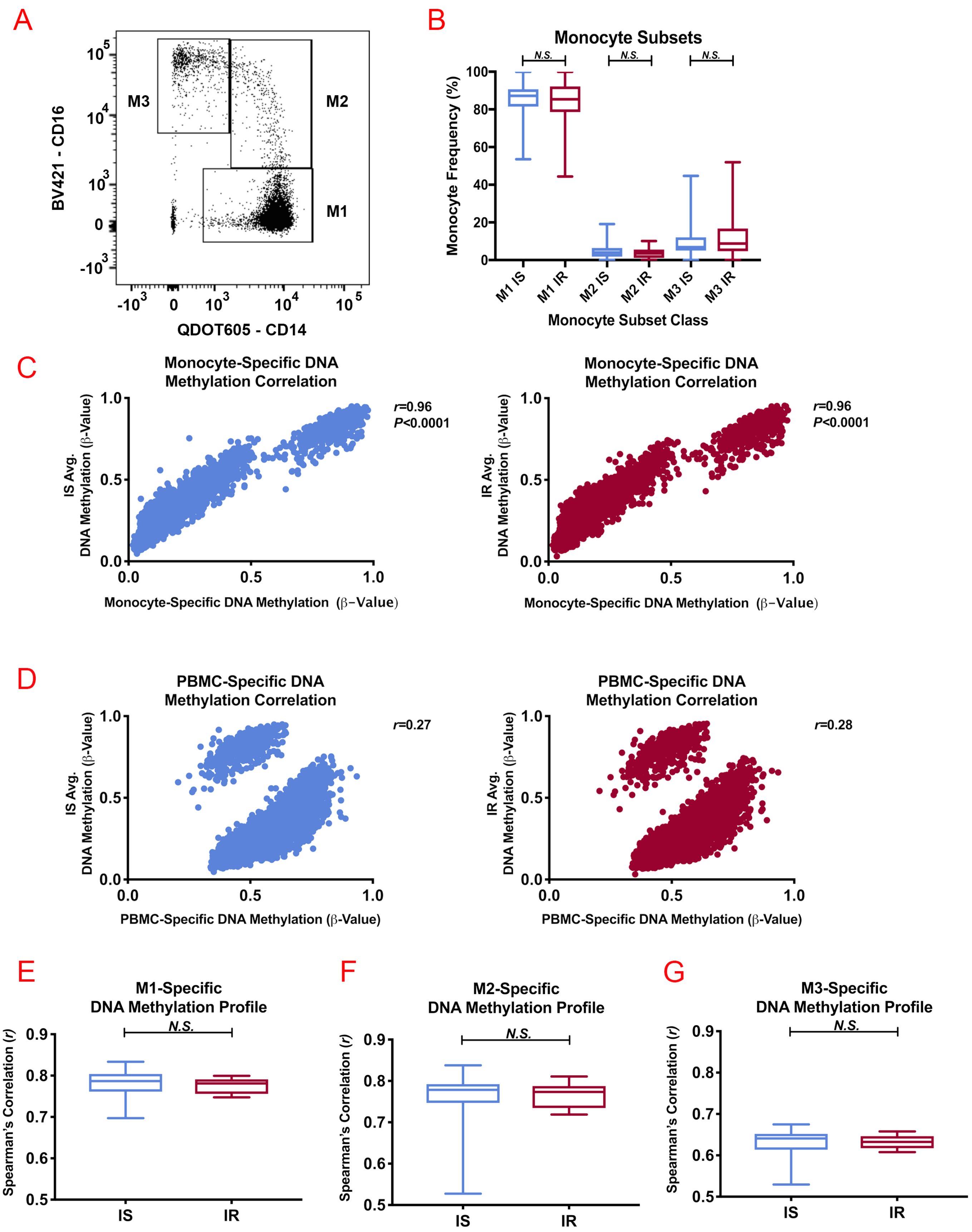
A
BV421 - CD16
QDOT605 - CD14
M3
M2
M1
B
Monocyte Subsets
Monocyte Frequency (%)
Monocyte Subset Class
M1 IS
M1 IR
M2 IS
M2 IR
M3 IS
M3 IR
N.S.
C
Monocyte-Specific DNA Methylation Correlation
IS Avg. DNA Methylation (β-Value)
IR Avg. DNA Methylation (β-Value)
Monocyte-Specific DNA Methylation (β-Value)
r=0.96
P<0.0001
D
PBMC-Specific DNA Methylation Correlation
PBMC-Specific DNA Methylation (β-Value)
r=0.27
r=0.28
E
M1-Specific DNA Methylation Profile
F
M2-Specific DNA Methylation Profile
G
M3-Specific DNA Methylation Profile
Spearman's Correlation (r)
IS
IR

Supplemental Figure 2. Independent confirmation of monocyte and monocyte subset composition by flow cytometry and DNA methylation analysis. A. Representative FACS analysis of gating strategy employed for determining monocyte subsets: M1(CD14++,CD16-), M2 (CD14++CD16+), and M3 (CD14+CD16++) monocytes. **B.** Frequency (%) of monocyte subsets was determined by cellular FACS-based phenotyping of monocytes from IS and IR individuals for classical (M1), intermediate (M2), and non-classical monocytes (M3); Significance at $P<0.05$ by Mann-Whitney U-test. N.S., non-significant. **C.** Linear regression analysis validates monocyte enrichment observed by flow cytometry using monocyte-specific DNA methylation data of FACS-sorted cells in comparison to our monocyte enrichment results from IS (blue) and IR (red) individuals as described in the methods section. **D.** Linear regression analysis of PBMC-specific DNA methylation profiles compared with monocytes enriched from IS (blue) and IR (red) individuals. Significance at $P<0.05$. Spearman's rho (*r*) was used to determine correlation coefficients. **E-G.** Correlation analysis of monocyte subset-specific DNA methylation profiles among IS and IR individuals. Spearman correlation coefficient values (*r*) were compared between each group for mean differences for indicated monocyte subsets (**E:** M1, **F**: M2, and **G:** M3); Significance at $P<0.05$.
