# Supplementary material for: Comparative DNA methylomic analyses reveal potential origins of novel epigenetic biomarkers of insulin resistance in monocytes from virally suppressed HIV-infected adults
Source: Clin Epigenetics. 2019 Jun 28;11:95. doi: 10.1186/s13148-019-0694-1 (PMC6599380; doi:10.1186/s13148-019-0694-1)
Supplement: Supplementary file 3 — Figure S3. Relationship between DNA methylation and HOMA-IR scores of DMLs enriched at genes involved in various insulin-related processes. DNA methylation derived from DMLs with their CpG probe ID shown that were enriched at genes involved in processes related to insulin signaling: MAPK11 (A), NSUFS7 (B), RAB1A (C), and CMKLR (D). The metylation levels at these CpGs in monocytes were significantly correlated with insulin resistance measured by HOMA-IR from IS (blue dots) and IR (red dots) individuals. Spearman correlation coefficient (r) shown with significance at P < 0.05. (PDF 9233 kb) [file 13148_2019_694_MOESM3_ESM.pdf]

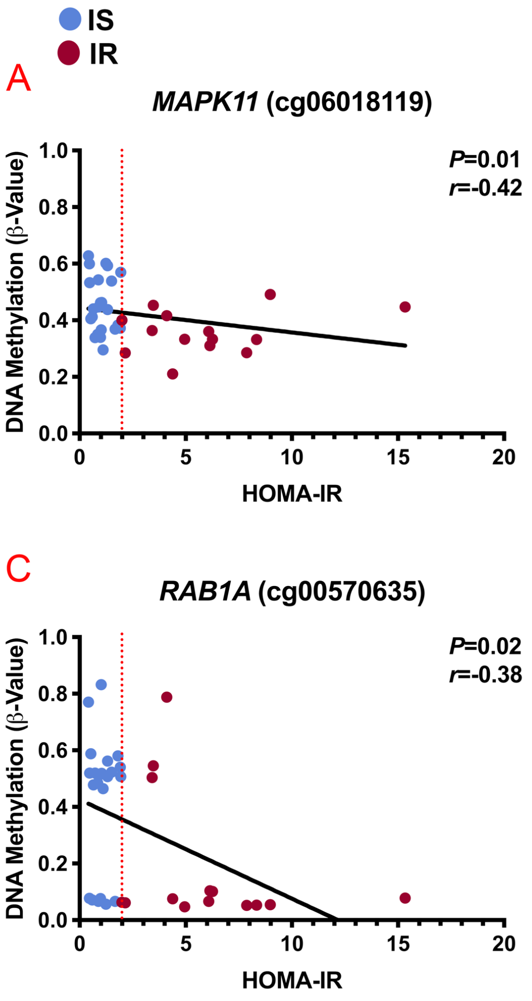


**Supplemental Figure 3. Relationship between DNA methylation and HOMA-IR scores of DMLs enriched at genes involved in various insulin-related processes.** DNA methylation derived from DMLs with their CpG probe ID shown that were enriched at genes involved in processes related to insulin signaling: *MAPK11* (**A**), *NSUFS7* (**B**), *RAB1A* (**C**), and *CMKLR* (**D**). The metylation levels at these CpGs in monocytes were significantly correlated with insulin resistance measured by HOMA-IR from IS (blue dots) and IR (red dots) individuals. Spearman correlation coefficient (r) shown with significance at $P<0.05$.
