# Supplementary material for: Comparative DNA methylomic analyses reveal potential origins of novel epigenetic biomarkers of insulin resistance in monocytes from virally suppressed HIV-infected adults
Source: Clin Epigenetics. 2019 Jun 28;11:95. doi: 10.1186/s13148-019-0694-1 (PMC6599380; doi:10.1186/s13148-019-0694-1)
Supplement: Supplementary file 4 — Figure S4. Differentially methylated loci from HIV-seronegative individuals stratified by HOMA-IR into IR or IS. A. Workflow for processing DNA methylation data acquired from 450k microarray to generate the HIV-seronegative DML of IR. B. Unsupervised hierarchal clustering heatmap using Manhattan distance, complete linkage method displays DNA methylation (β value) of the 304 CpGs of the HIV-seronegative DML of IR, stratifies IS individuals (green) from IR individuals (purple). DNA methylation ranges from low (0, blue) to intermediate (.50, yellow), to high (1.0, red). C. Vendiagram is representative of the two datasets that culminate the DML of IR in HIV-infected individuals (left; DML of IR) and HIV-seronegative individuals (right; HIV-seronegative DML of IR). Shown are the CpG counts that are independent to each dataset and the CpGs that are overlapped in both datasets; below displays the number of genes harbored at the CpGs. D. Comparison of monocyte subset-specific DNA methylation correlation of HIV-seronegative IR and IS individuals. Correlation determined utilizing Spearman’s rho (r); significance determined using Mann-Whitney U test between IS and IR at P < 0.05. (PDF 9233 kb) [file 13148_2019_694_MOESM4_ESM.pdf]

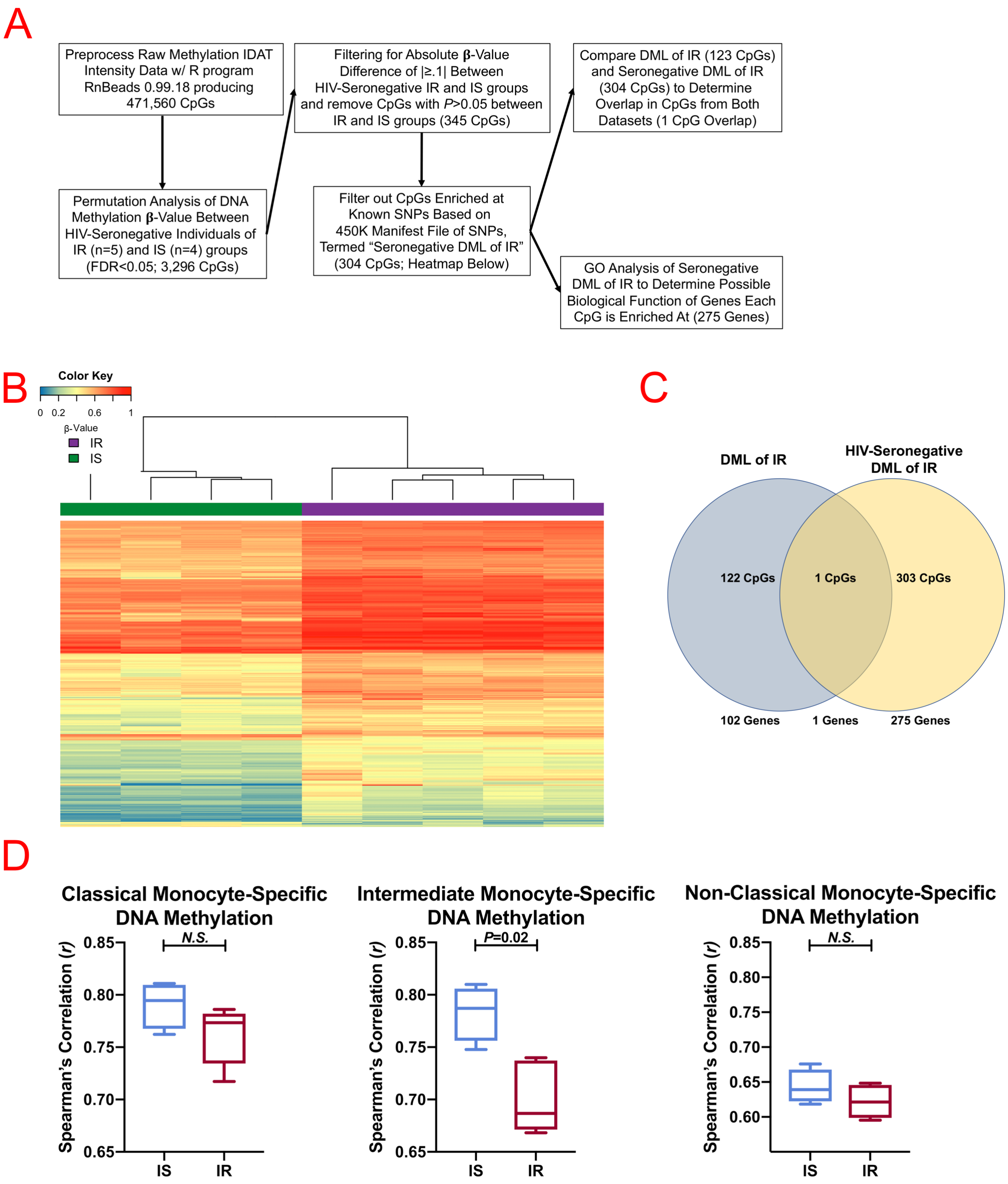

Supplemental Figure 4. Differentially methylated loci from HIV-seronegative individuals stratified by HOMA-IR into IR or IS. A. Workflow for processing DNA methylation data acquired from 450k microarray to generate the HIV-Seronegative DML of IR. **B.** Unsupervised hierarchal clustering heatmap using Manhattan distance, complete linkage method displays DNA methylation (β-value) of the 304 CpGs of the HIV-Seronegative DML of IR, stratifies IS individuals (Green) from IR individuals

(Purple). DNA methylation ranges from low (0, blue) to intermediate (.50, yellow), to high (1.0, red). **C.** Vendiagram is representative of the two datasets that culminate the DML of IR in HIV-infected individuals (left; DML of IR) and HIV-Seronegative individuals (right; HIV-Seronegative DML of IR). Shown are the CpG counts that are independent to each dataset and the CpGs that are overlapped in both datasets; below displays the number of genes harbored at the CpGs. **D.** Comparison of monocyte subset-specific DNA methylation correlation of HIV-Seronegative IR and IS individuals. Correlation determined utilizing Spearman's rho *(r)*; significance determined using Mann-Whitney U-test between IS and IR at $P<0.05$.
