# Supplementary material for: Comparative DNA methylomic analyses reveal potential origins of novel epigenetic biomarkers of insulin resistance in monocytes from virally suppressed HIV-infected adults
Source: Clin Epigenetics. 2019 Jun 28;11:95. doi: 10.1186/s13148-019-0694-1 (PMC6599380; doi:10.1186/s13148-019-0694-1)
Supplement: Supplementary file 5 — Table S3. Clinical characteristics of HIV-seronegative individuals stratified by insulin sensitivity and insulin resistance. Table represents clinical data comparing the IS and IR groups of HIV-seronegative individuals. Data shown are median values [first quartile, third quartile]. P value determined between IS and IR groups using Mann-Whitney U test; significance at P < 0.05. (PDF 9233 kb) [file 13148_2019_694_MOESM5_ESM.pdf]

Supplemental Table 3. HIV-Seronegative Clinical Characteristics			
	IS (n=4)	IR (n=5)	*P* -value
Age	47.50 [42.25 , 58.00]	59.00 [51.00 , 64.00]	0.11
Gender (Male; n)	2	3	-
Diabetic (n)	-	3	-
Systolic Blood Pressure (mmHg)	106.00 [105.30 , 109.80]	119.00 [117.00 , 129.50]	0.01
Total Cholesterol (mg/dL)	196.50 [164.30 , 249.80]	185.00 [158.00 , 207.00]	0.56
HDL Cholesterol (mg/dL)	63.50 [57.25 , 78.75]	51.00 [38.50 , 58.00]	0.05
HOMA-IR	0.88 [0.73 , 1.20]	3.36 [3.00 , 3.71]	-
FRS	0.01 [0.01 , 0.01]	0.20 [.020 , 0.21]	0.02

Supplemental Table 3. Clinical characteristics of HIV-Seronegative individuals stratified by insulin sensitive and insulin resistance. Table represents clinical data comparing the IS and IR groups of HIV-Seronegative individuals. Data shown are median values [1st quartile , 3rd quartile]. *P*-value determined between IS and IR groups using Mann-Whitney U-test; significance at $P<0.05$.
