# Supplementary material for: Comparative DNA methylomic analyses reveal potential origins of novel epigenetic biomarkers of insulin resistance in monocytes from virally suppressed HIV-infected adults
Source: Clin Epigenetics. 2019 Jun 28;11:95. doi: 10.1186/s13148-019-0694-1 (PMC6599380; doi:10.1186/s13148-019-0694-1)
Supplement: Supplementary file 6 — Table S2. GO analysis of HIV-seronegative DML of IR. Table displays significant biological functions of the DMLs enriched at genes involved in immune responses, metabolic processes, and signaling pathways. Included in table are GO analysis P value calculated using the Fisher exact test (P < 0.05), the CpG probe ID, genomic position of the DML, the gene(s) harboring each CpG, the genomic location of the CpG in the context of a gene, the mean DNA methylation in both the HIV-seronegative IS and IR groups, the difference in methylation (Delta Value), and the P value calculated between the IS and IR groups as determined using the Mann-Whitney U test and taken at a significance of P < 0.05. (PDF 9233 kb) [file 13148_2019_694_MOESM6_ESM.pdf]

Supplemental Table 2. GO Analysis of Seronegative DML of IR									
**Biological Process**	**GO *P*-value**	**Probe ID**	**Genomic Position**	**Gene(s)**	**Gene Region**	**Mean IS**	**Mean IR**	**Delta Value**	***P*-Value**
antigen processing and presentation of exogenous peptide antigen via MHC class I	0.005	*cg14479884*	chr7:80267943-80267944	*CD36*	5'UTR	0.24	0.48	0.24	0.02
	0.005	*cg11741432*	chr17:4696249-4696250	*PSMB6*	Intergenic (Upstream)	0.55	0.69	0.14	0.02
	0.005	*cg15193228*	chr22:30855332-30855333	*SEC14L3*	3'UTR	0.66	0.56	-0.10	0.02
	0.005	*cg07166546*	chr14:35805898-35805899	*PSMA6*	Intergenic (Downstream)	0.46	0.63	0.17	0.02
	0.005	*cg20149362*	chr1:7834809-7834810	*VAMP3*	Body	0.63	0.74	0.11	0.02
regulation of cAMP metabolic process	0.02	*cg26367013*	chr14:93385970-93385971	*CHGA*	Intergenic (Upstream)	0.71	0.83	0.12	0.06
	0.02	*cg06908855*	chr7:93201042-93201043	*CALCR*	5'UTR	0.56	0.69	0.13	0.02
hexose transport	0.02	*cg00648005*	chr1:9086914-9086915	*SLC2A7*	TSS1500	0.50	0.61	0.11	0.03
	0.02	*cg00500507*	chr22:32439391-32439392	*SLC5A1*	1st Exon	0.42	0.60	0.17	0.03
	0.02	*cg03604322*	chr22:3243052-3243053	*SLC5A1*	5'UTR	0.46	0.62	0.16	0.02
leukocyte adhesion to vascular endothelial cell	0.02	*cg04972065*	chr12:53591766-53591767	*ITGB7*	Body	0.24	0.35	0.11	0.02
	0.02	*cg02303209*	chr5:32223673-32223674	*GOLPH3*	Intergenic (Upstream)	0.62	0.73	0.11	0.02
positive regulation of Wnt signaling pathway	0.05	*cg15451548*	chr1:2144873-2144874	*SKI*	Intergenic (Upstream)	0.47	0.57	0.10	0.02
	0.05	*cg11741432*	chr17:4696249-4696250	*PSMB6*	Intergenic (Upstream)	0.55	0.69	0.14	0.02
	0.05	*cg03961481*	chr16:51180521-51180522	*SALL1*	Body	0.25	0.44	0.19	0.02
	0.05	*cg17166338*	chr5:1295969-1295970	*TERT*	TSS1500	0.72	0.84	0.12	0.02
	0.05	*cg07166546*	chr14:35805898-35805899	*PSMA6*	Intergenic (Downstream)	0.46	0.63	0.17	0.02
response to glucocorticoid	0.05	*cg17744604*	chr1:206946166-206946167	*IL10*	TSS1500	0.17	0.36	0.19	0.02
	0.05	*cg06908855*	chr7:93201042-93201043	*CALCR*	5'UTR	0.56	0.69	0.13	0.02
regulation of glucose metabolic process	0.05	*cg18865445*	chr3:110522265-110522266	*IRS2*	Intergenic (Upstream)	0.31	0.49	0.18	0.03
	0.05	*cg14211930*	chr9:97402452-97402453	*FBP1*	5'UTR	0.11	0.21	0.10	0.02

Supplemental Table 2. GO analysis of HIV-Seronegative DML of IR. Table displays significant biological functions of the DMLs enriched at genes involved in immune responses, metabolic processes, and signaling pathways. Included in table are GO analysis *P*-value calculated using the Fisher Exact test ($P<0.05$)**,** the CpG probe ID, genomic position of the DML, the gene(s) harboring each CpG, the genomic location of the CpG in the context of a gene, the mean DNA methylation in both the HIV-seronegative IS and IR groups, the difference in methylation (Delta Value), and the *P*-value calculated between the IS and IR groups as determined using the Mann-Whitney U-Test and taken at a significance of $P<0.05$.
