# Supplementary material for: Comparative DNA methylomic analyses reveal potential origins of novel epigenetic biomarkers of insulin resistance in monocytes from virally suppressed HIV-infected adults
Source: Clin Epigenetics. 2019 Jun 28;11:95. doi: 10.1186/s13148-019-0694-1 (PMC6599380; doi:10.1186/s13148-019-0694-1)
Supplement: Supplementary file 7 — Table S1. Datasets used for DNA methylation analyses in this study. List of the GEO accession numbers for the datasets used for DNA methylation analyses incorporated into study. (PDF 9233 kb) [file 13148_2019_694_MOESM7_ESM.pdf]

Supplemental Table 1. Datasets Used for DNA Methylation Analysis	
**Description**	**GEO Accession**
Total Monocyte-Specific DNA Methylation	GSE35069
PBMC-Specific DNA Methylation	GSE35069
Monocyte Subset-Specific DNA Methylation	GSE131627
HIV-Seronegative DNA Methylation	GSE131627
HIV+ IR/IS DNA Methylation	GSE131627
HSC DNA Methylation	GSE87197

Supplemental Table 1. Datasets used for DNA methylation analyses in this study. List of the GEO accession numbers for the datasets used for DNA methylation analyses incorporated into study.
